# An ESP32-S3 Multimodal Wearable Sensor Platform with Embedded XGBoost for Focal-to-Bilateral Tonic–Clonic Seizure Detection and IoT Alerting

**DOI:** 10.3390/s26216710

**Published:** 2026-10-23

**Authors:** Mohamed Ali Torad, Mina Hany Thabet, Mohammed Youssef Taha, Ahmed Ali Mohamed Torad, Mona Mohamed Taha, András Attila Horváth, Mohamed Abdelaziz Emam

**Affiliations:** 1 Department of Electronics and Communication Engineering, Higher Technological Institute (HTI), 10th of Ramadan City 44634, Egypt; minahany925@gmail.com (M.H.T.); mohammedfazza402@gmail.com (M.Y.T.); 2 Department of Physical Therapy and Health Rehabilitation, Jouf University, Sakaka 72341, Saudi Arabia; almtorad@ju.edu.sa; 3 Department of Basic Sciences, Faculty of Physical Therapy, Kafrelsheikh University, Kafrelsheikh 33516, Egypt; 4 Department of Rehabilitation Sciences, College of Health and Rehabilitation Sciences, Princess Nourah bint Abdulrahman University, P.O. Box 84428, Riyadh 11671, Saudi Arabia; momtaha@pnu.edu.sa; 5 Neurocognitive Research Centre, Nyírő Gyula National Institute of Psychiatry and Addictology, 1085 Budapest, Hungary; andras.horvath.semmelweis@gmail.com; 6 Department of Anatomy, Histology and Embryology, Semmelweis University, 1085 Budapest, Hungary; 7 János Szentágothai Neurosciences Division, Semmelweis University, 1085 Budapest, Hungary

**Keywords:** wearable sensors, epilepsy, seizure detection, focal-to-bilateral tonic–clonic seizure, electromyography, accelerometer, edge AI, XGBoost, ESP32-S3, internet of things

## Abstract

**Highlights:**

**What are the main findings?**

- A multimodal wearable prototype performs focal-to-bilateral tonic–clonic seizure detection entirely on an ESP32-S3 edge platform, using synchronized 10 s EMG and accelerometer windows converted to 26 statistical features and classified by an embedded 85-tree XGBoost model.
- Embedded and Python inference were numerically equivalent to within 1.19 × 10^−7^ in probability output.
- Across a reported validation record spanning 2643.69 h, 12/18 record-labeled FBTCS events were detected (67% event sensitivity), with a reported false-alarm rate of 0.31 per 24 h and a 61.5 s median detection latency.

**What are the implications of the main findings?**

- The engineering prototype demonstrates the feasibility of fully embedded, on-device multimodal seizure detection.
- Prospective clinical validation and improved sensitivity and latency are required before medical deployment.

**Abstract:**

Wearable seizure-detection systems can provide objective event monitoring and rapid caregiver alerting without continuous bedside observation. This study presents a resource-constrained multimodal wearable sensor platform for focal-to-bilateral tonic–clonic seizure (FBTCS) detection using edge artificial intelligence and Internet of Things (IoT) communication. The prototype integrates an ESP32-S3 microcontroller, an analog electromyography (EMG) channel, an MPU6050 inertial sensor, MAX30102 photoplethysmography, GPS, a TFT display, local vibration/buzzer outputs, and Blynk cloud telemetry. The seizure–decision path uses synchronized EMG and acceleration acquired at 100 Hz in non-overlapping 10 s windows. EMG and acceleration are resampled for compatibility with the reference feature pipeline, 13 statistical features are extracted from each modality (26 total), and an 85-tree XGBoost model is executed locally. A probability threshold of 0.85 is combined with temporal smoothing, consecutive-window confirmation, and a 60 s refractory interval. Software verification showed maximum absolute probability differences of approximately 1.19 × 10^−7^ for random feature-vector tests and 1.49 × 10^−8^ for end-to-end recorded windows relative to the Python reference. Within the reported validation record covering 2643.69 h and 18 record-labeled FBTCS events, 12 events were detected (12/18; 67% sensitivity). Precision was approximately 26%, F1-score approximately 38%, the reported false-alarm rate was 0.31 per 24 h, and median detection latency was 61.5 s. Approximately 34 false alarms are estimated from the reported false-alarm rate and evaluation duration; this is an estimated count rather than a percentage or directly observed count. Hardware, local alarm, display, GPS, and cloud communication functions passed functional integration tests. The small event count, uncertain label provenance, low precision, and long latency mean that these data support an engineering proof-of-concept only and do not establish clinical applicability.

## 1. Introduction

Epilepsy is a chronic neurological disorder affecting approximately 50 million people worldwide [1]. Beyond the neurological manifestations themselves, unpredictability of seizures creates risks of injury and contributes to substantial limitations in daily activity. Objective detection is also clinically relevant because patient- or caregiver-reported seizure diaries can be incomplete, particularly for nocturnal or unwitnessed events. Wearable digital health technologies therefore have an important potential role in event logging, alerting, and longitudinal assessment [2].

Wearable seizure detectors are most mature for generalized tonic–clonic and focal-to-bilateral tonic–clonic seizures because these events often produce strong motor and autonomic signatures that can be captured outside conventional electroencephalography (EEG). The International League Against Epilepsy and International Federation of Clinical Neurophysiology clinical practice guideline supports clinically validated wearable devices for automated detection of tonic–clonic seizures in appropriate patients, while emphasizing the need for robust validation and acceptable false-alarm burden [3]. A systematic review and meta-analysis likewise found high mean sensitivity for tonic–clonic seizure detection but highlighted heterogeneity and false alarms as persistent limitations [4].

Several sensing modalities have been investigated. Surface electromyography (EMG) can capture sustained tonic and clonic muscle activation; accelerometry and gyroscopes capture rhythmic motor patterns; electrodermal activity and photoplethysmography (PPG) provide autonomic context; and reduced-channel wearable EEG provides information closer to the cerebral source. Beniczky et al. demonstrated real-time tonic–clonic seizure detection using wearable surface EMG [5]. Kusmakar et al. reported high sensitivity with a wrist-worn accelerometer approach [6]. Multimodal wrist systems combining accelerometry and electrodermal activity have also been evaluated across multicenter and prospective studies [7,8,9]. More recently, Zhang et al. showed that adding wearable EEG to EMG and accelerometry can reduce false positives while maintaining high sensitivity [10].

The emergence of open, long-duration multimodal datasets supports reproducible algorithm development. SeizeIT2 is a multicenter wearable dataset of patients with focal epilepsy that includes behind-the-ear EEG, ECG, EMG, accelerometry, and gyroscope recordings across more than 11,000 h [11]. Such datasets enable algorithms to be trained and evaluated independently of a particular embedded hardware implementation, after which a compact feature set and classifier can be transferred to a wearable edge device.

Edge execution is attractive because local inference remains available when internet connectivity is absent, can reduce communication latency, and limits the amount of raw physiological data that must be continuously transmitted. The ESP32-S3 provides dual-core processing, integrated Wi-Fi/Bluetooth Low Energy, on-chip memory, and common peripheral interfaces in a resource-constrained microcontroller platform [12]. XGBoost is well suited to compact tabular feature vectors because tree ensembles provide nonlinear decision boundaries while allowing deterministic inference with modest computational resources [13].

This study evaluates the proposed system as a multimodal wearable FBTCS detector with embedded edge intelligence and IoT alerting. The specific contributions are: (i) a complete sensing-to-alert architecture integrating EMG, inertial sensing, PPG, GPS, local outputs, and IoT telemetry; (ii) a deterministic 100 Hz acquisition and 10 s feature-inference pipeline on an ESP32-S3; (iii) embedded deployment of an 85-tree XGBoost classifier using 26 statistical EMG/acceleration features; (iv) numerical verification against the Python[x.x] reference implementation; and (v) descriptive event-level reporting from the available validation record covering 2643.69 h using sensitivity, precision, F1-score, false alarms per 24 h, and detection latency. The event-level record is used to characterize detector behavior and does not constitute prospective clinical validation of the custom wearable. Importantly, the reported evidence supports seizure detection and alerting, not prospective pre-ictal seizure prediction.

## 2. Literature Review

### 2.1. Wearable Seizure Detection: Clinical Requirements and Overall Evidence

Wearable seizure-detection research has concentrated primarily on generalized tonic–clonic seizures and focal-to-bilateral tonic–clonic seizures because these events generate prominent motor, muscular, and autonomic signatures. The ILAE/IFCN clinical practice guideline concluded that automated wearable detection can be clinically useful for tonic–clonic seizures when devices are appropriately validated, while also emphasizing the importance of false-alarm burden, detection delay, and independent clinical evaluation [3]. A systematic review and meta-analysis of noninvasive wearable systems reported a mean tonic–clonic seizure sensitivity of approximately 91% and an overall false-alarm rate of 2.1 per 24 h, demonstrating that high sensitivity is achievable but that nuisance alarms remain a major practical limitation [4]. These findings provide an important benchmark for positioning the proposed system.

To strengthen the empirical basis of the review, the studies below are discussed using their reported cohort/event sizes and operating outcomes rather than by device description alone. The review distinguishes retrospective/algorithm-development evidence from prospective clinical validation and from embedded engineering deployment, because these evidence levels answer different questions about accuracy, generalization, and real-world feasibility.

### 2.2. Surface-EMG Detection: Beniczky et al. [5]

Beniczky et al. evaluated a wearable surface-EMG device for automated real-time detection of generalized tonic–clonic seizures [5]. The device exploited the characteristic high-amplitude, high-frequency muscular activity of tonic–clonic events and reported 93.8% sensitivity with a false-alarm rate of 0.67 per day; the study also reported detection latency within 30 s. The work demonstrated that a compact single-modality sensor can provide strong detection performance. Its principal limitations are dependence on reliable skin–electrode contact and the fact that a single muscle channel may be vulnerable to placement variability, vigorous non-seizure activity, or events with atypical motor expression. The proposed system retains EMG as a core decision modality but supplements it with accelerometry and an embedded edge-processing architecture.

### 2.3. Wrist Accelerometry: Kusmakar et al. [6]

Kusmakar et al. developed a wrist-worn accelerometer approach using time-domain and Poincaré-plot-derived motion features followed by kernelized support vector data description [6]. Evaluation covered 5576 h from 79 patients. Across several convulsive-event types the algorithm detected 40 of 46 events, while the generalized tonic–clonic seizure subgroup achieved 95.23% sensitivity with 0.64 false alarms per 24 h. The principal advantage is unobtrusive sensing with a single wrist device; however, accelerometry alone can confuse vigorous activities of daily living with convulsive motion. This limitation motivates multimodal fusion, including the EMG-plus-acceleration decision path used in the proposed system.

### 2.4. Multimodal Wrist Sensing: Onorati et al. [7]

Onorati et al. investigated a multimodal wrist-worn detector combining three-axis accelerometry with electrodermal activity [7]. The multicenter evaluation detected 52 of 55 convulsive seizures, corresponding to approximately 94.5% event sensitivity, while producing about one false alarm every five days (approximately 0.2 per 24 h). Combining motion with autonomic information reduced the ambiguity inherent in movement-only sensing and showed the value of multimodal confirmation. The trade-off is greater sensor and algorithm complexity than unimodal systems. The Empatica wristband literature further established this accelerometry-plus-electrodermal-activity architecture as an important platform for ambulatory seizure monitoring [9].

### 2.5. Prospective Fixed-Algorithm Validation: Onorati et al. [8]

A later prospective multicenter study by Onorati et al. evaluated a fixed-and-frozen accelerometry-plus-electrodermal-activity algorithm in 152 patients over 429 monitoring days, including 66 convulsive seizures [8]. Corrected sensitivity was 0.92 in pediatric participants and 0.94 in adults. False-alarm rates were 1.26 and 0.57 per 24 h, respectively. An alternative more specific operating mode reduced the false-alarm rate by 68% while maintaining 0.95 sensitivity across the population. This study is particularly important because it used independent patients and expert video–EEG reference labeling, illustrating the level of prospective validation expected for clinical translation. The proposed system has not yet reached this validation level.

### 2.6. Wearable EEG, EMG, and Accelerometry Fusion: Zhang et al. [10]

Zhang et al. evaluated behind-the-ear wearable EEG together with EMG and accelerometry in 27 participants who experienced 44 tonic–clonic seizures [10]. Wearable EEG alone provided very high sensitivity but also generated a comparatively high false-positive rate. Fusion improved the balance between sensitivity and false alarms: the EEG-plus-EMG combination achieved 97.7% sensitivity with 0.4 false positives per 24 h, while the three-modality EEG-plus-EMG-plus-accelerometry configuration achieved 90.9% sensitivity, 0.1 false positives per 24 h, 75.5% precision, and an 82.5% F1-score. This work shows that complementary cerebral, muscular, and motion information can substantially improve discrimination, although the additional wearable EEG hardware and signal processing increase implementation complexity relative to the proposed microcontroller-based platform.

### 2.7. SeizeIT2 Dataset and Research Gap

The SeizeIT2 dataset described by Bhagubai et al. provides an important foundation for reproducible wearable seizure research [11]. It contains approximately 11,640 h of wearable recordings and includes behind-the-ear EEG, ECG, EMG, accelerometry, and gyroscope signals from patients with focal epilepsy. The dataset enables algorithm development without requiring every research group to create a new clinical acquisition cohort. However, successful algorithm development on a public dataset does not by itself validate a custom wearable device, because electrode characteristics, sensor placement, analog front ends, motion artifacts, sampling behavior, and patient populations can differ. The proposed system therefore addresses an engineering gap—transferring a compact EMG/accelerometry classifier to a standalone ESP32-S3 platform with local alarms, display, GPS context, and IoT telemetry—but still requires prospective clinical validation of the custom hardware.

#### Recent Edge-AI and Microcontroller Seizure-Detection Implementations

Recent work has moved seizure detection closer to the extreme edge. Qian et al. mapped a compressed-learning seizure detector to an MSP432 MCU and reported a 50.13 kB memory requirement, 13.4727 mW power consumption, 269.4 μJ per classification, and 21.07 ms classification latency [14]. Ingolfsson et al. developed BrainFuseNet for wearable EEG–PPG–accelerometer fusion and evaluated deployment on a GAP9 ultra-low-power microcontroller, reporting 0.11 mJ per inference at its high-performance operating point [15]. Hizem et al. presented a TinyML workflow for EEG seizure detection with an optimized model size of 256 kB for microcontroller-class hardware [16]. These studies show that embedded seizure detection can be characterized using resource, energy, model-size, and latency metrics in addition to clinical detection metrics. In comparison, the present work contributes an ESP32-S3 implementation of a compact 85-tree XGBoost decision path with local IoT alerting, but the present validation record does not contain measured power, battery runtime, RAM/flash utilization, or inference-time benchmarks; therefore, the engineering claim is limited to functional edge feasibility rather than demonstrated resource superiority.

To broaden this comparison, four further edge-AI seizure-detection implementations were considered (Table 1). Busia et al. designed EEGformer, a compact transformer for temporal-channel EEG, and deployed it on the Apollo4, GAP8, and GAP9 microcontrollers; the GAP9 implementation required 13.7 ms and 0.31 mJ per inference, with 73% seizure detection and 0.15 false positives per hour on the CHB-MIT dataset [17]. Li et al. deployed a spiking neural network on the Xylo neuromorphic processor and reported an average power consumption of 87.4 μW (input/output) plus 287.9 μW (compute) [18]. Zanghieri et al. implemented a temporal convolutional network for intracranial EEG on a low-power parallel MCU, requiring 5.68 ms and 124.5 μJ per inference on one core and 1.46 ms and 51.2 μJ on eight cores [19]. Popa et al. deployed EEG classifiers on an ESP32 microcontroller (520 KiB RAM, 240 MHz) and reported a 15 ms inference time for an int8-quantized seizure model [20]; this is the closest platform-level comparator to the ESP32-S3 used here. Two observations follow from this comparison. First, nearly all recent edge implementations target EEG and are evaluated on benchmark datasets at the window or recording level, whereas the proposed system targets the motor signature of FBTCS using EMG and acceleration and reports event-level metrics over long-term recordings; detection metrics are therefore not directly comparable across these studies. Second, these studies report per-inference latency and energy measured on the target hardware, which the present work does not yet provide. Section 3.6.6 therefore gives an analytical estimate of the computational load of the proposed pipeline, and measured profiling on the ESP32-S3 is identified as required future work.

### 2.8. Comparative Analysis and Position of the Proposed System

Table 2 summarizes the main reviewed systems and places the proposed system in the same context. The comparison shows a clear progression from single-modality EMG or accelerometry toward multimodal sensor fusion and rigorous prospective validation. The proposed system differs primarily in its emphasis on microcontroller-class embedded execution: feature extraction, XGBoost inference, temporal confirmation, local alarming, and IoT communication are integrated on the ESP32-S3. Its reported false-alarm rate is comparatively low, but its 67% event sensitivity and 61.5 s median detection latency are substantially weaker than the best clinically evaluated systems. Accordingly, the contribution of the present work is engineering feasibility and edge integration rather than state-of-the-art clinical detection accuracy.

Sensor location is an important confounder in this comparison. The proposed prototype uses upper-arm surface-EMG electrodes, whereas several comparator systems are wrist-worn and sense acceleration, electrodermal activity, PPG, or other peripheral signals. Upper-arm EMG directly reflects activity of the instrumented muscle and is sensitive to electrode contact, orientation, skin impedance, and muscle recruitment; wrist signals instead emphasize distal motion and autonomic responses. Consequently, differences in wear location and modality can change signal amplitude, artifact exposure, feature distributions, and event detectability. The literature values in Table 2 used as contextual benchmarks and not as evidence of location-independent performance equivalence.

## 3. Materials and Methods

### 3.1. System Scope and Design Requirements

The proposed system was developed as a wearable research platform for focal-to-bilateral tonic–clonic seizure (FBTCS) detection and caregiver alerting. The design objective was to combine synchronized physiological and motion sensing, embedded machine-learning inference, local audiovisual/haptic alarms, graphical feedback, and IoT telemetry in a self-contained ESP32-S3 platform. The final seizure–decision pathway uses EMG and acceleration-derived features, while photoplethysmography (PPG), gyroscope measurements, and GPS are retained as contextual channels. The device is an engineering prototype and is not intended for diagnosis, treatment decisions, or unsupervised emergency deployment.

The implementation requirements included deterministic 100 Hz acquisition of EMG and inertial signals, bounded inference time, robust sensor initialization and recovery, explicit signal-quality states, local operation after firmware upload, and remote status transmission through Blynk. Estimated GPS coordinates used when a valid satellite fix was unavailable were visibly identified as estimated rather than verified emergency locations.

### 3.2. Development Workflow

**Figure 1:**
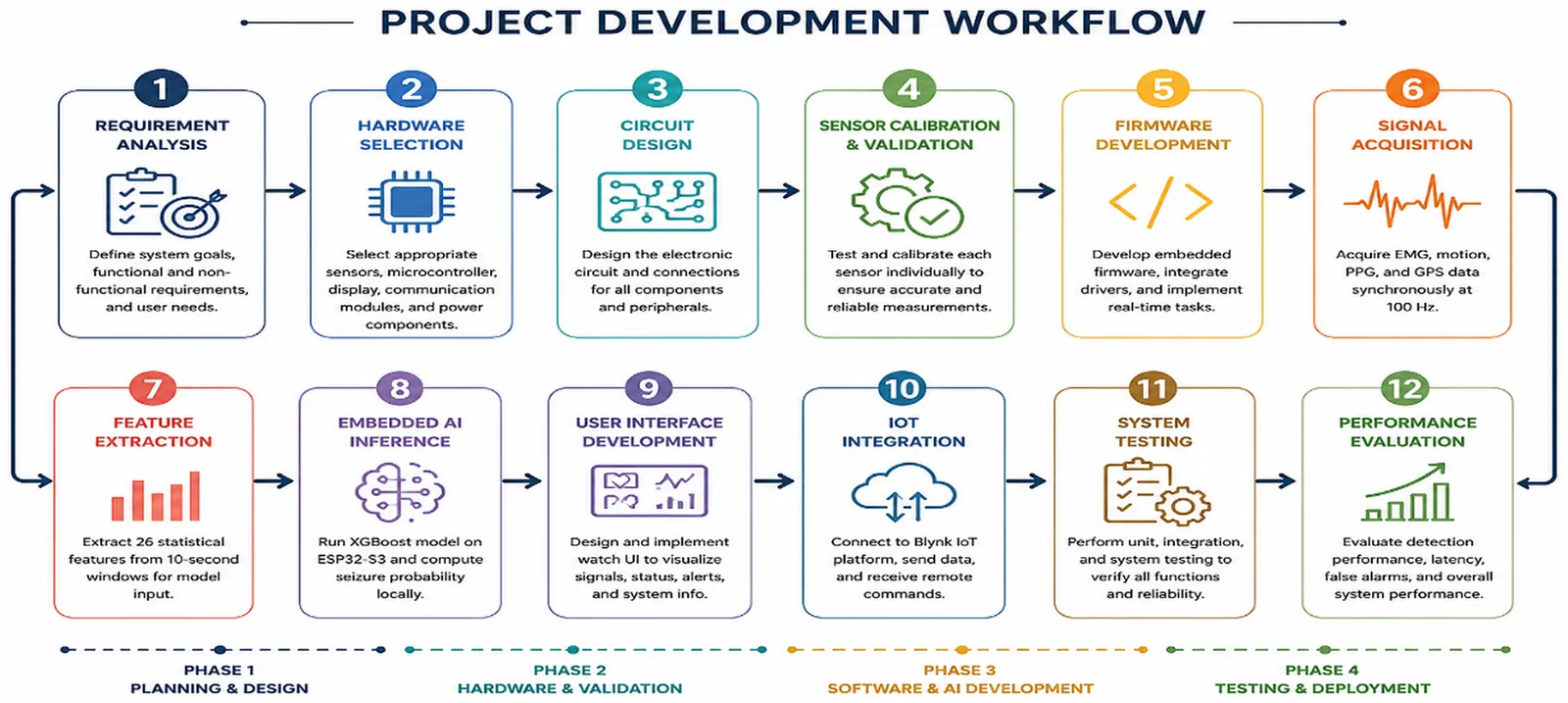
Development workflow of the proposed system, from requirements analysis and hardware validation through embedded-AI integration, IoT implementation, testing, and performance evaluation.

Development followed a staged engineering workflow in which requirements analysis and hardware selection preceded circuit design, sensor calibration, firmware implementation, synchronized signal acquisition, feature extraction, embedded AI integration, user-interface development, IoT integration, system testing, and performance evaluation. This sequence reduced debugging ambiguity by verifying individual subsystems before complete integration. Figure 1 illustrates the complete staged development workflow.

### 3.3. System Development Methodology

**Figure 2:**
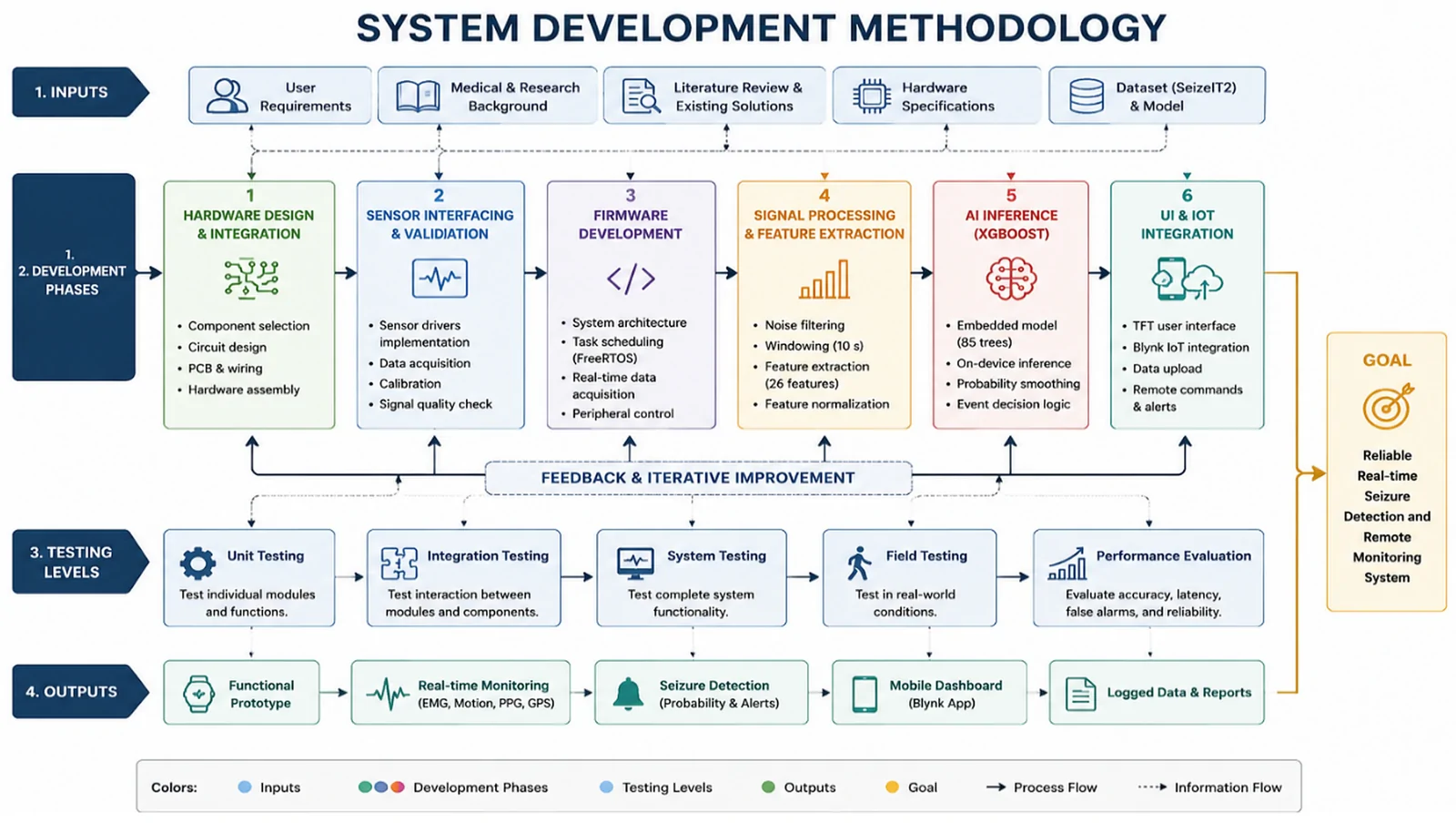
System development methodology showing the principal implementation phases, testing layers, feedback loops, and final engineering outputs.

The development methodology was organized around iterative implementation and feedback. Inputs from the user, medical literature, available wearable datasets, hardware limitations, and supervisor requirements were translated into hardware, firmware, AI, interface, and validation phases. Testing results were fed back into earlier stages so that sensor initialization, signal quality, display behavior, and alarm logic could be refined before final system evaluation. The iterative methodology and its feedback paths are summarized in Figure 2.

### 3.4. System Architecture and Hardware Design

#### 3.4.1. Overall System Architecture

**Figure 3:**
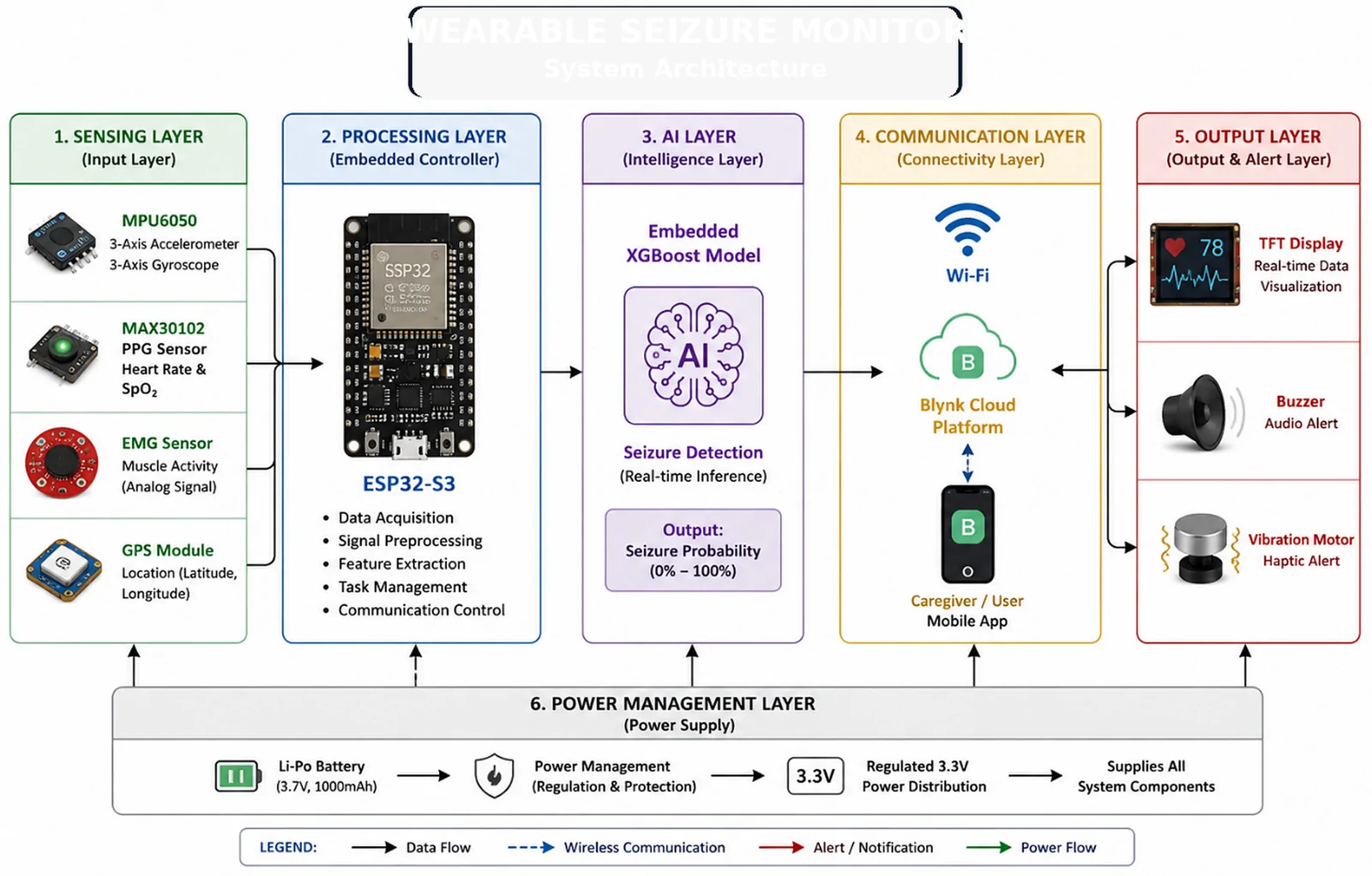
Overall architecture of the proposed wearable platform, showing sensing, ESP32-S3 processing, embedded AI, communication, output, and power-management layers.

The proposed system is organized into sensing, processing, embedded-AI, communication, output, and power-management layers. The ESP32-S3 acts as the central controller [12]. EMG is acquired through an analog biopotential front end, motion is measured by the MPU6050 inertial sensor [21], heart-rate/PPG context is obtained using the MAX30102 [22], and location is acquired through a NEO-6-series GPS module [23]. The processor performs feature extraction and XGBoost inference locally, while the display, buzzer, and vibration motor provide immediate feedback and Wi-Fi/Blynk provides remote telemetry [24,25]. Figure 3 presents the overall system architecture, and Table 3 summarizes the principal hardware components, interfaces, and functions used in the prototype.

#### 3.4.2. Hardware Block Diagram and Circuit-Level Implementation

The hardware block diagram emphasizes signal routing and interface compatibility. Analog EMG enters the ESP32-S3 ADC, the MPU6050 and MAX30102 share the I^2^C bus, the GPS receiver uses a hardware UART, and the TFT display is driven through SPI. Buzzer and vibration outputs are controlled through driver stages so that alarm actuation does not load the microcontroller pins directly.

For EMG acquisition, the project hardware uses an AD8232-based single-lead biopotential front end whose analog output is connected to ESP32-S3 GPIO4. The signal must remain within the ESP32-S3 0–3.3 V ADC range. In the final schematic, the AD8232 lead-off pins are not routed to the ESP32-S3; therefore, EMG validity is assessed in firmware using saturation count and window-range checks rather than LO+/LO− lead-off logic. EMG and acceleration are acquired synchronously at 100 Hz into 10 s windows before feature processing. These details are reported to make the target-hardware acquisition chain explicit.

The conventional resonant-sensor quality factor (Q) is not a meaningful single performance parameter for this mixed sensing chain (AD8232 biopotential front end, MPU6050 inertial sensor, and MAX30102 optical sensor). Sensor quality is therefore described through acquisition validity and calibration checks: ADC range/saturation and EMG variation, stationary acceleration and gyroscope bias, PPG signal-quality gating, and GPS data reception.

**Figure 4:**
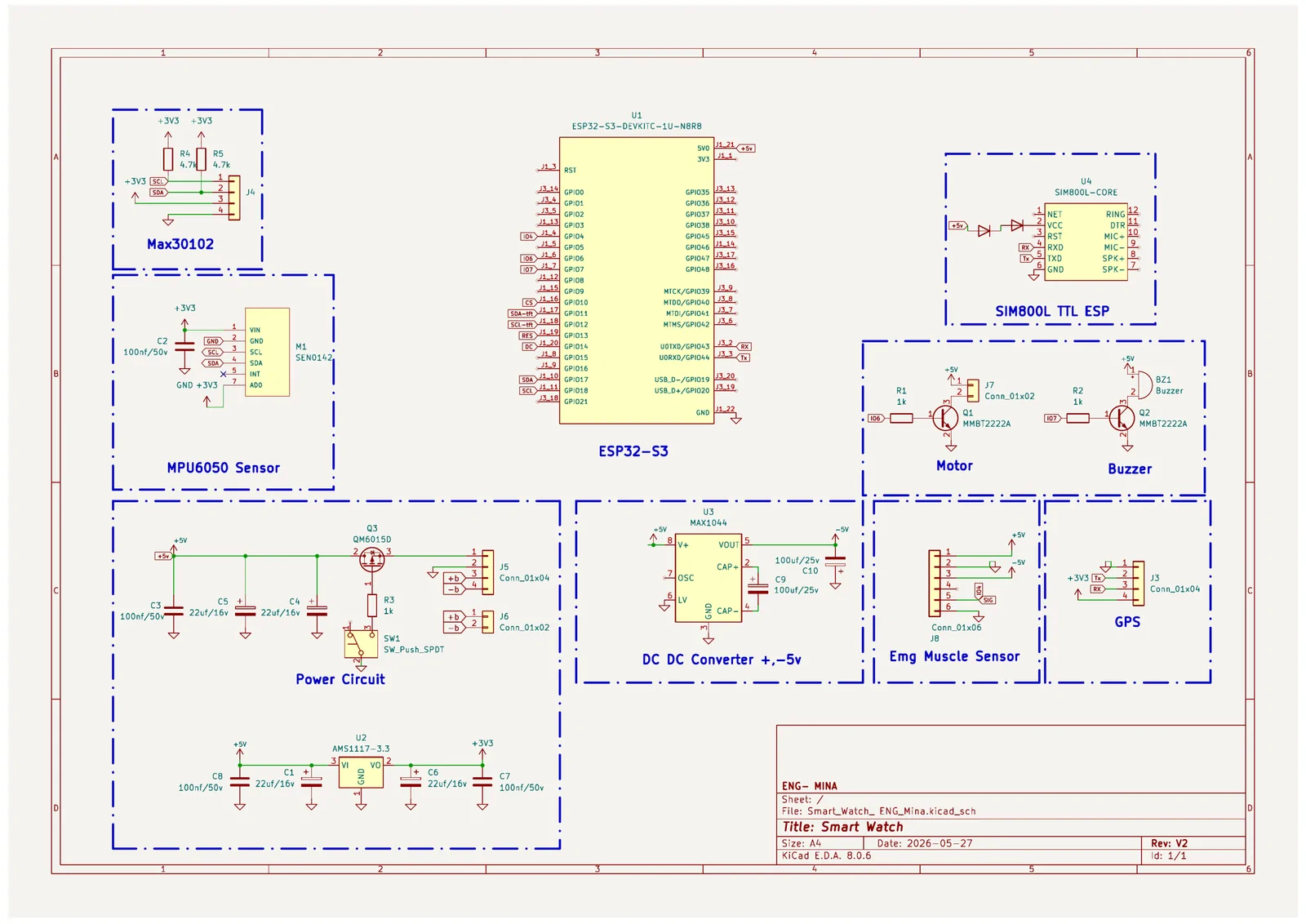
Circuit-level hardware schematic used for integration of the proposed wearable prototype.

The circuit-level schematic documents the physical interconnection of the controller, sensing modules, display, alarm outputs, and supporting power circuitry. The schematic was used as the wiring reference during prototype integration and hardware troubleshooting. The implemented circuit-level interconnection is shown in Figure 4.

#### 3.4.3. Hardware Selection Rationale and Limitations

Hardware selection in this prototype was implementation-driven rather than the result of a formal sensor-optimization study. The project documentation supports electrical/interface compatibility with the ESP32-S3, available firmware/library integration, and successful subsystem testing, but it does not document a head-to-head comparison against alternative EMG front ends, newer inertial sensors, or other PPG devices. Accordingly, the selected components should be interpreted as practical prototype choices rather than an optimized seizure-sensing architecture.

The AD8232-based channel was used as a single-lead biopotential conditioning stage with an analog output connected directly to ESP32-S3 GPIO4. This simplified prototype integration, but the device is a general single-lead biopotential/heart-rate front end rather than a dedicated research-grade EMG front end. The final hardware does not route the AD8232 lead-off pins to the microcontroller, the analog output must remain within the 0–3.3 V ADC range, and electrode contact, motion artifact, placement, and the uncharacterized analog bandwidth/gain can influence the acquired EMG. These limitations restrict direct equivalence with the reference dataset.

The MPU6050 is a mature six-axis I^2^C inertial sensor and was retained because it was already integrated into the prototype acquisition and calibration workflow. The project record contains no systematic benchmark against newer IMUs, so no claim is made that it is the optimal motion sensor for seizure detection. The MAX30102 supplies red/infrared PPG samples for heart-rate estimation, signal-quality gating, display, and IoT telemetry. In this manuscript, “contextual” therefore means that PPG is monitored and reported but does not contribute a feature to the seizure classifier or confirm/cancel an alarm. No completed experiment comparing EMG + ACC against EMG + ACC + PPG was present in the source record; any benefit from adding PPG to the classifier remains unproven and requires a formal ablation study.

#### 3.4.4. Effective Sensor Modalities

To remove ambiguity about which sensors determine the seizure decision, Table 4 separates the sensing channels by their functional role. Only two modalities are effective inputs to the classifier: single-lead EMG and acceleration magnitude, each contributing 13 of the 26 features. The gyroscope, PPG, and GPS channels are acquired, displayed, and transmitted, but none of them provides a classifier feature or can confirm, suppress, or cancel an alarm. The system is therefore a bimodal EMG + ACC detector housed in a multisensor platform, and any benefit of the contextual channels for detection performance remains untested.

### 3.5. Embedded Software and Real-Time Firmware

#### 3.5.1. Software Workflow

**Figure 5:**
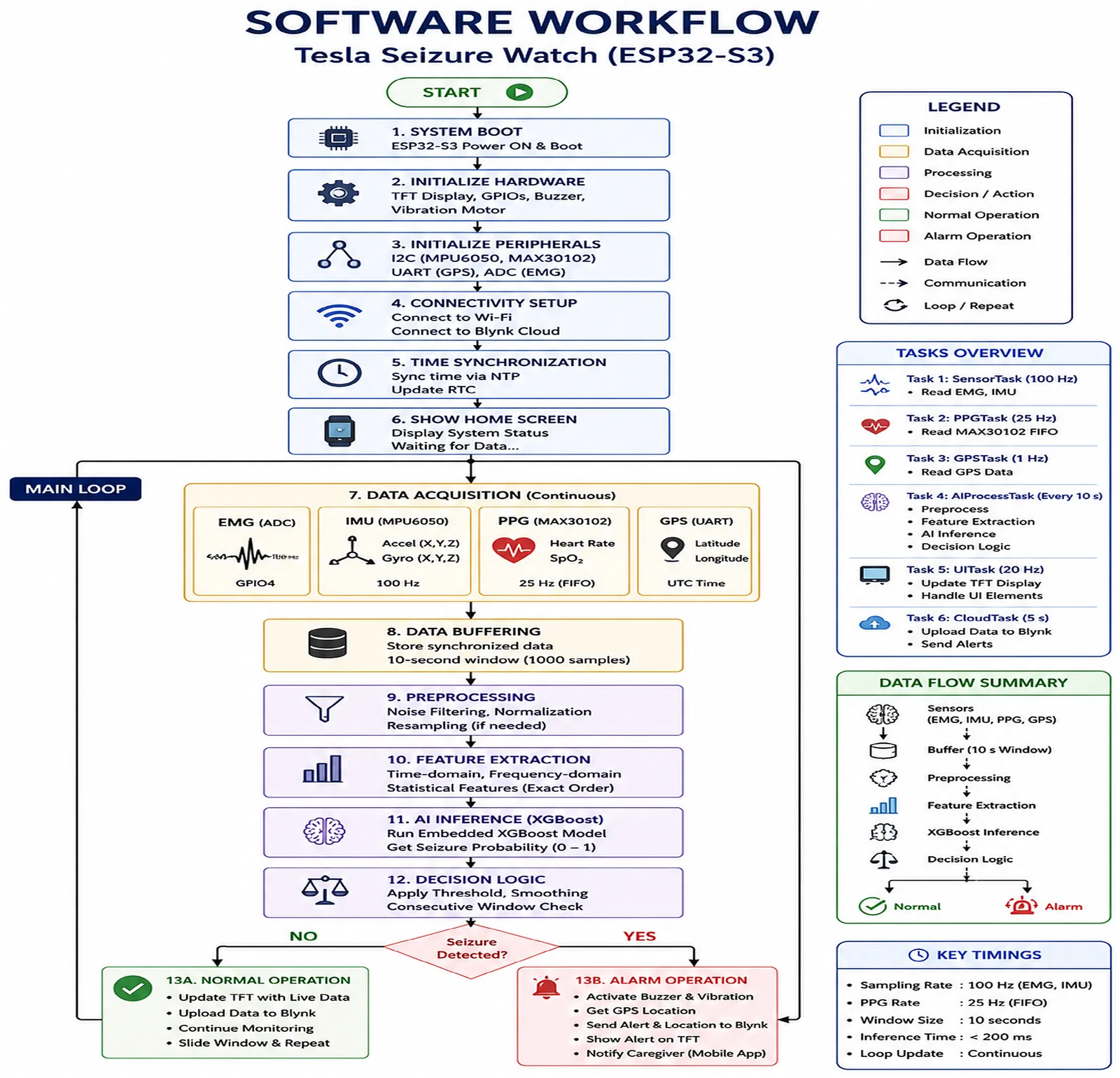
Software workflow of the proposed system, including initialization, sensing, preprocessing, embedded inference, alarm handling, interface updates, telemetry, and fault management.

The embedded software was implemented in C/C++ using the Arduino framework. Initialization is followed by sensor acquisition, data-quality checks, buffering, feature extraction, embedded inference, alarm-state evaluation, graphical-interface updates, GPS processing, and Blynk telemetry. Error states are handled explicitly so that missing or invalid sensor data are not silently treated as valid measurements. Figure 5 summarizes this embedded software workflow.

#### 3.5.2. Firmware Architecture and Scheduling

**Figure 6:**
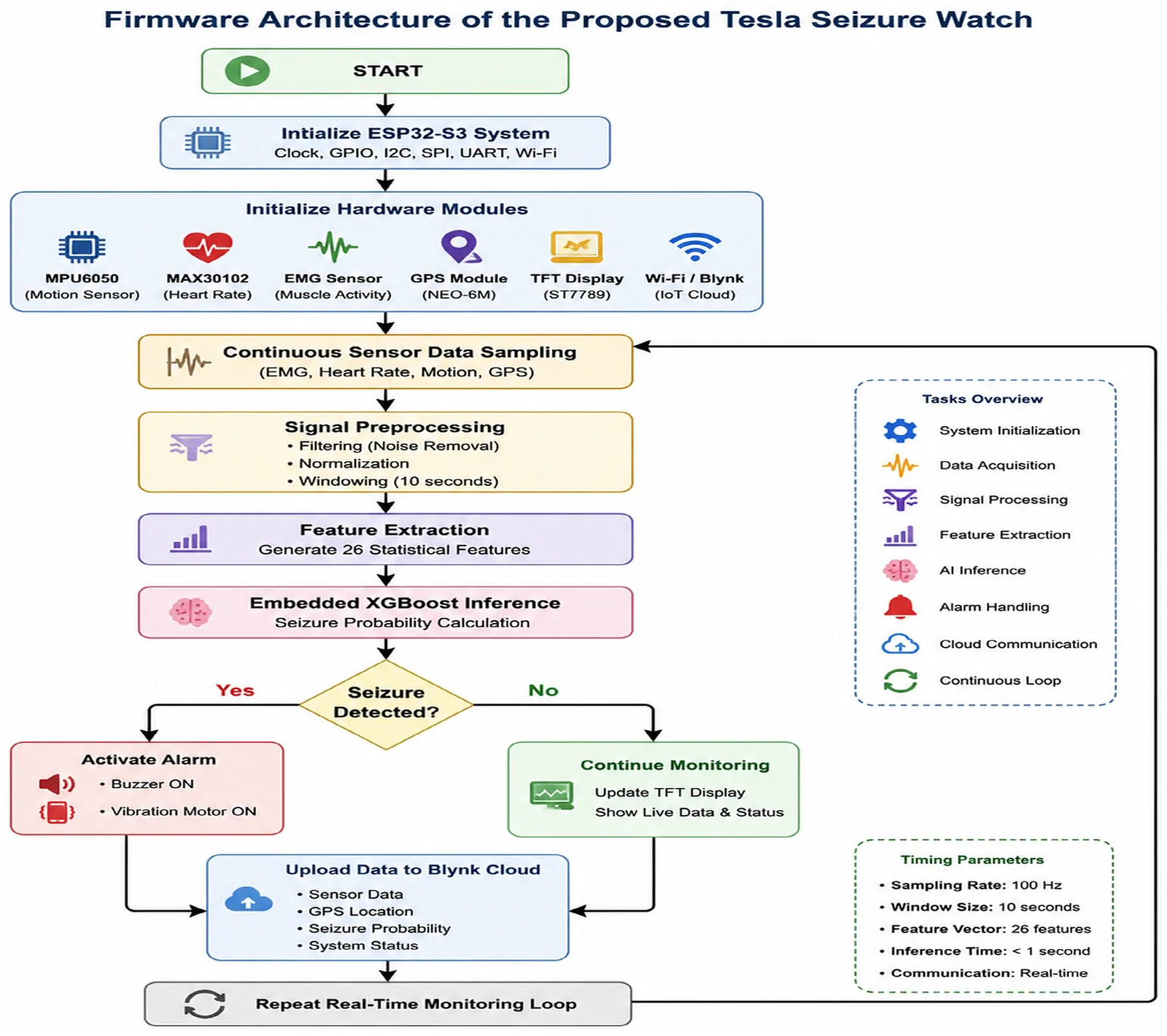
Firmware architecture of the proposed system, showing initialization, concurrent acquisition, buffering, AI inference, display management, IoT communication, and alarm control.

FreeRTOS scheduling [27] was used to separate time-critical acquisition from slower interface and network operations. EMG and acceleration were sampled synchronously at 100 Hz. Two 1000-sample buffers were alternated so that acquisition could continue while the completed 10 s window was processed. PPG samples were serviced from the MAX30102 FIFO, the user interface was refreshed at approximately 200 ms intervals, and Blynk telemetry was updated at approximately 1 s intervals. Figure 6 illustrates the firmware architecture, while Table 5 summarizes the principal data paths, execution rates, and functions.

#### 3.5.3. On-Device Interface and Alarm Output Verification

**Figure 7:**
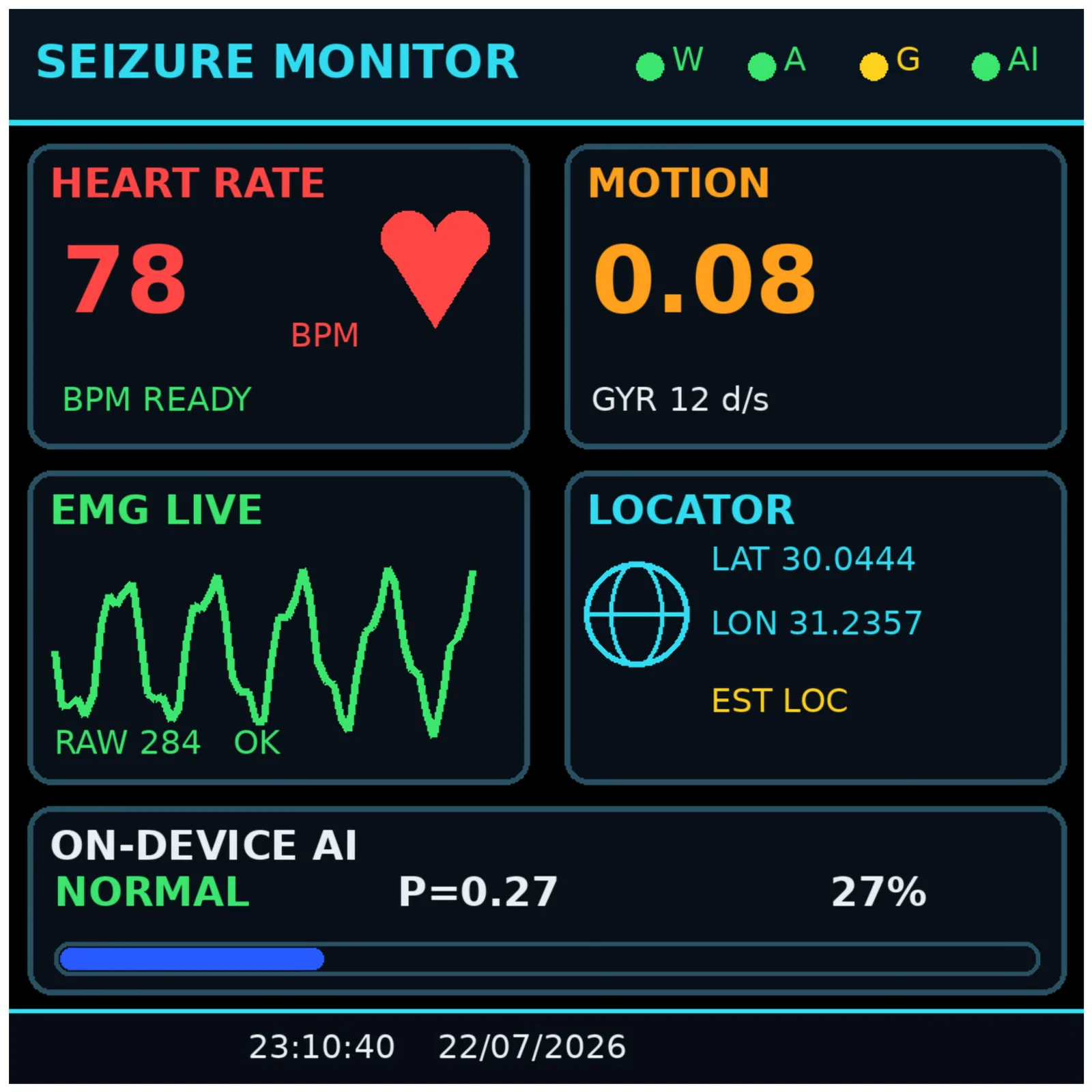
On-device graphical interface showing physiological status, motion, EMG activity, locator state, connectivity indicators, and embedded-AI probability.

**Figure 8:**
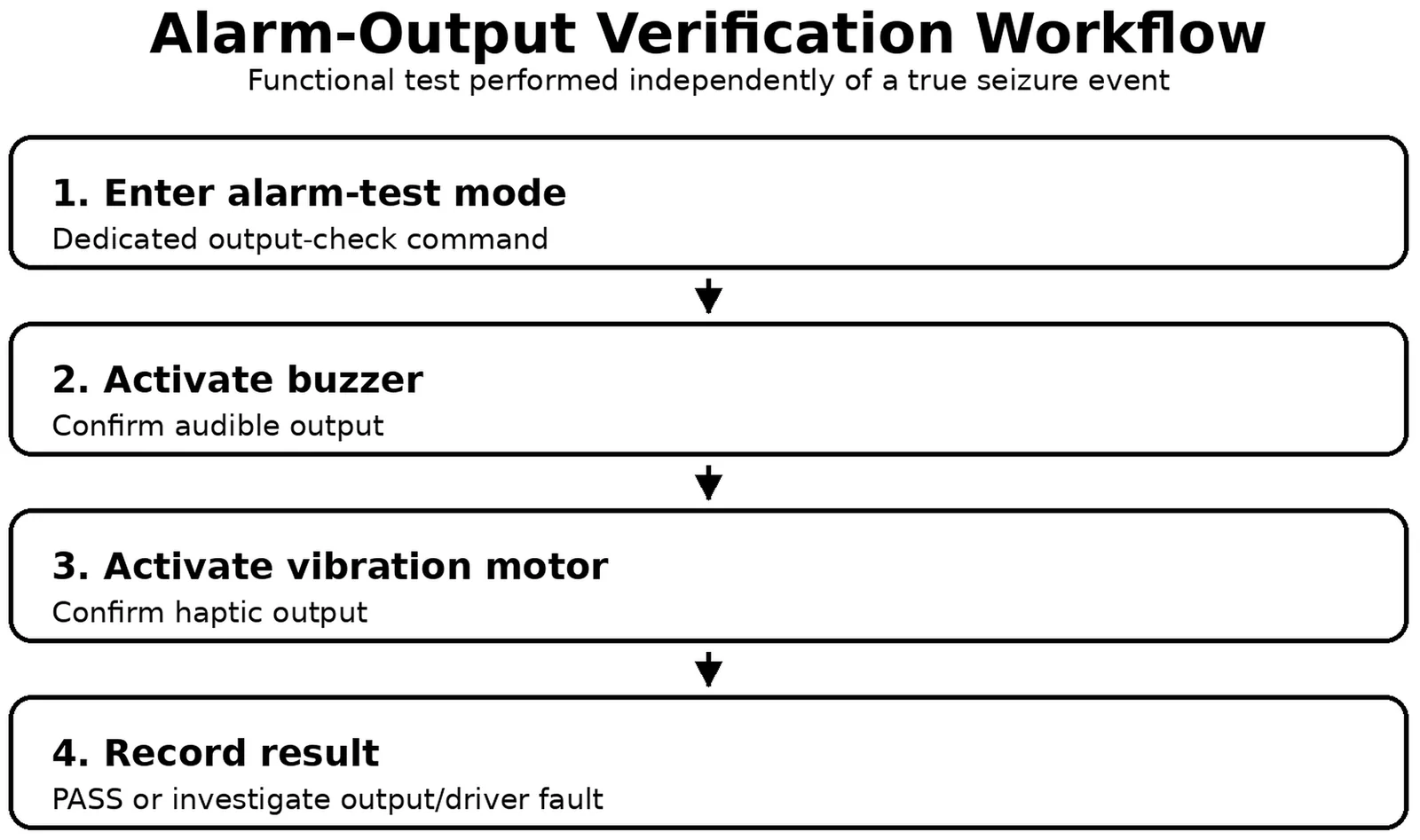
Alarm-output verification workflow used to test buzzer and vibration activation independently of a true seizure event.

The TFT interface presents heart rate, motion, live EMG behavior, location state, connectivity indicators, AI probability/state, date/time, and alarm status. Normal operation avoids unnecessary full-screen redraws to preserve responsiveness. A dedicated alarm-test mode verifies the local output path before deployment. The normal on-device interface is shown in Figure 7, whereas Figure 8 now presents the alarm-output verification sequence in a readable functional workflow.

Any historical internal development label visible in prototype screen captures is only a laboratory user-interface label; it is not presented as a commercial product name, trademark, or commercialization claim.

### 3.6. Embedded AI Model and Signal Processing

#### 3.6.1. Dataset, Windowing, and Feature Extraction

Model development used the SeizeIT2 wearable dataset [11], which contains long-duration multimodal recordings from patients with focal epilepsy. For the embedded seizure–decision path, EMG and acceleration magnitude were processed in 10 s windows. Hardware EMG and inertial data were acquired at 100 Hz. Before statistical summarization, EMG samples were interpolated to 256 Hz and acceleration was resampled to 25 Hz to maintain compatibility with the reference feature-extraction interface.

The 100-to-256 Hz EMG operation is an interpolation step only and does not restore frequency content above the 50 Hz Nyquist limit of the 100 Hz hardware acquisition. Accordingly, native higher-rate dataset EMG and 100 Hz custom-hardware EMG should not be assumed to be spectrally equivalent. This sampling-domain difference is a potential source of dataset-to-device domain shift and is considered when interpreting transfer of the SeizeIT2-derived classifier to the prototype.

A controlled sampling-frequency experiment was not present in the supplied source record. Therefore, the manuscript does not claim a measured performance penalty from the 100 Hz hardware rate. The expected mechanism is instead stated explicitly: 100 Hz acquisition limits recoverable EMG content to below 50 Hz, while subsequent interpolation changes sample density but cannot recreate lost high-frequency information. A future sampling ablation should train/evaluate matched pipelines at native and hardware-limited rates to quantify the change in sensitivity, false-alarm burden, and latency.

Thirteen time-domain statistics were calculated independently for EMG and acceleration magnitude, resulting in a 26-element feature vector. The selected descriptors include mean, standard deviation, centered RMS, median, interquartile range, extrema, range, absolute-deviation measures, difference RMS, line length, and zero-crossing rate. The compact feature representation was selected to keep memory and computational requirements compatible with microcontroller-class edge inference. Table 6 lists the complete statistical feature set used for both EMG and acceleration magnitude.

The 13 descriptors were inherited from the existing trained feature pipeline and were retained to preserve exact compatibility with the exported XGBoost model; the available record does not contain a separate feature-ranking or redundancy-pruning study. Several descriptors overlap conceptually. In particular, centered RMS and standard deviation are mathematically redundant when the same population normalization is used, while difference RMS and line length both summarize successive-sample variation. Therefore, the manuscript does not claim that all 26 inputs are independently informative or optimally selected. A future retraining study should quantify inter-feature correlation and model importance and compare pruned feature sets using a fixed patient-level validation protocol.

For completeness, the 13 statistics are defined formally as follows. Let *x*_1_, …, *x*_n_ denote the samples of one 10 s window after resampling, where *N* = 2560 for EMG (256 Hz) and *N* = 250 for acceleration magnitude (25 Hz), and acceleration magnitude is *a*_i_ = √(*a*_x,i_^2^ + *a*_y,i_^2^ + *a*_z,i_^2^). With mean μ = (1/*N*)Σ*x*_i_ and centered samples *c*_i_ = *x*_i_ − μ, the features are: standard deviation σ = √[(1/*N*)Σ*c*_i_^2^]; centered RMS = √[(1/*N*)Σ*c*_i_^2^]; median; interquartile range IQR = *Q*_75_ − *Q*_25_; minimum, maximum, and range = max − min; mean absolute deviation = (1/*N*)Σ|*c*_i_|; maximum absolute deviation = max_i_|*c*_i_|; difference RMS = √[(1/(*N* − 1))Σ(*x*_i+1_ − *x*_i_)^2^]; line length = (1/(*N* − 1))Σ|*x*_i+1_ − *x*_i_|; and zero-crossing rate = (1/(*N* − 1))Σ𝟙[*c*_i_·*c*_i+1_ < 0]. The EMG and acceleration feature sets are concatenated in a fixed order to form the 26-element input vector expected by the exported model.

#### 3.6.2. XGBoost Decision Flow

**Figure 9:**
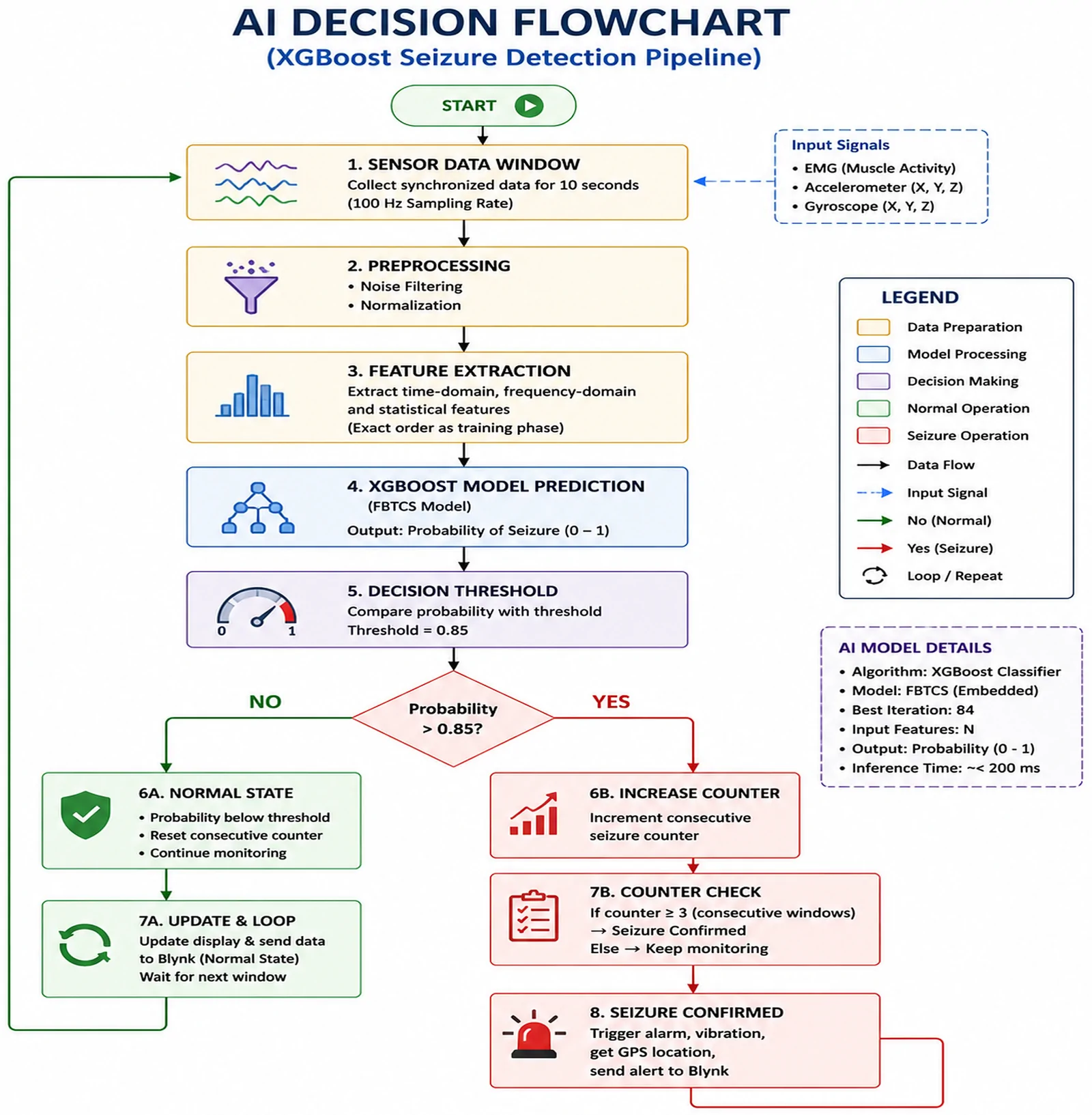
AI decision flow implemented in the proposed system, from synchronized sensor windows and signal preprocessing through feature extraction, XGBoost inference, temporal decision logic, and alarm generation.

The classifier was implemented with XGBoost [13]. The source model contained 165 trees and identified best iteration 84; consequently, the embedded package retained trees 0–84 (85 trees total). No retraining occurs on the ESP32-S3; the firmware executes the exported tree ensemble. At run time, the firmware checks signal validity, calculates the 26 features, traverses the stored decision trees, accumulates the model margin, and converts the margin to a seizure probability using a logistic transformation. Figure 9 illustrates the complete XGBoost decision flow from validated features to the final seizure-probability output.

Formally, for a feature vector x ∈ ℝ^26^, each tree *f*_t_ maps x to the value of the leaf reached by comparing, at each internal node, one feature with its stored split threshold (following the XGBoost convention of taking the left branch when the feature value is below the threshold). The embedded model output is the margin *m*(x) = *b*_0_ + Σ_t=0_^84^ *f*_t_(x), where *b*_0_ is the base margin stored in the exported model, and the window-level seizure probability is *p* = 1/(1 + e^(−*m*(x))). Because windows that fail the signal-validity checks are not passed to the classifier, missing-value default branches are not exercised during normal operation.

The exported source model metadata identifies best_iteration = 84, which explains why trees 0–84 were embedded. However, neither the manuscript source record nor the main project thesis documents the validation metric, early-stopping patience, complete XGBoost hyperparameters, class-balancing procedure, random seed, or model-selection rule that made iteration 84 optimal. The value is therefore reported as exported-model metadata, not as an independently reproduced optimization result. Full reproduction of model development requires disclosure of these training details together with the patient-level data split.

#### 3.6.3. Embedded Processing and On-Device Detection Pipeline

**Figure 10:**
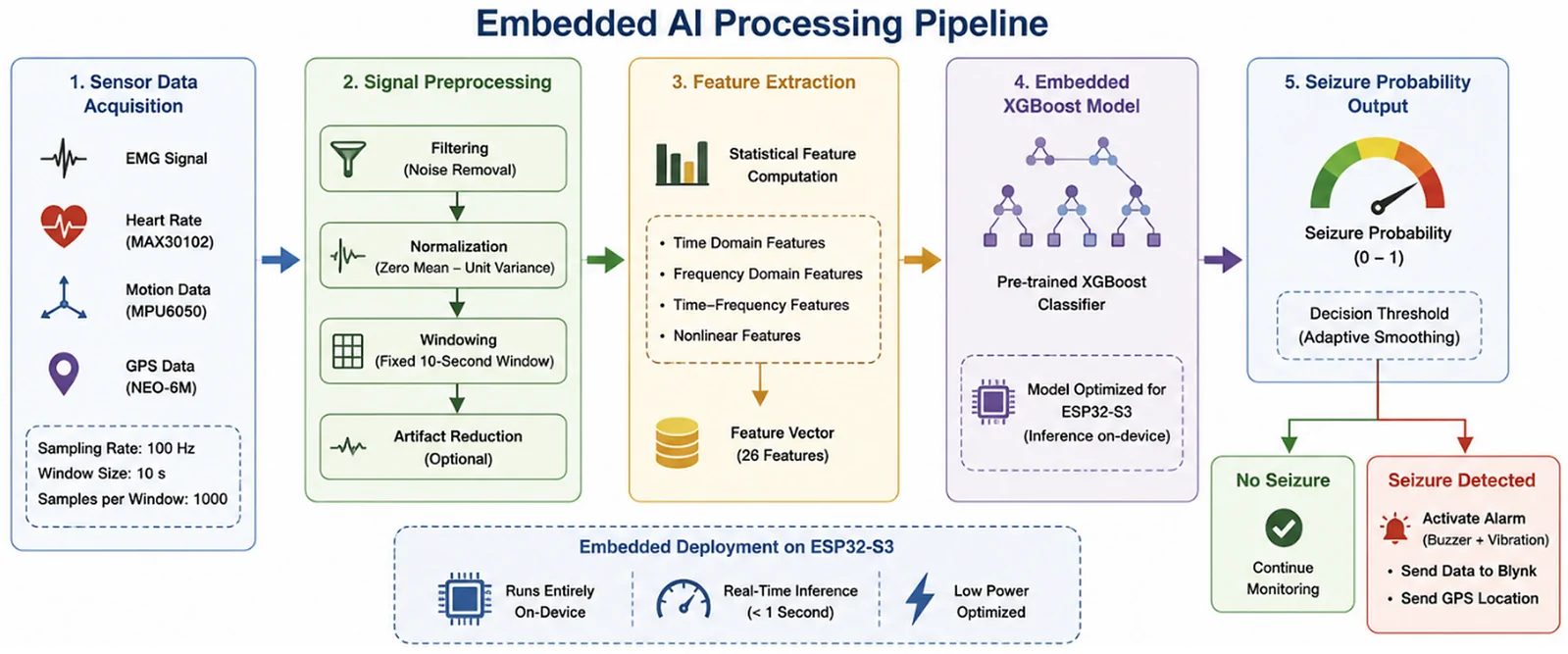
Embedded AI processing pipeline, including sensor acquisition, signal preprocessing, statistical feature extraction, XGBoost inference, seizure-probability generation, and alarm decision.

The end-to-end embedded processing chain separates sensing and preprocessing from the classifier and final alarm logic. This separation is important because the probabilistic model output is not itself an alarm: the probability is subsequently smoothed and subjected to consecutive-window confirmation to reduce isolated false activations. Figure 10 summarizes this embedded processing pipeline.

**Figure 11:**
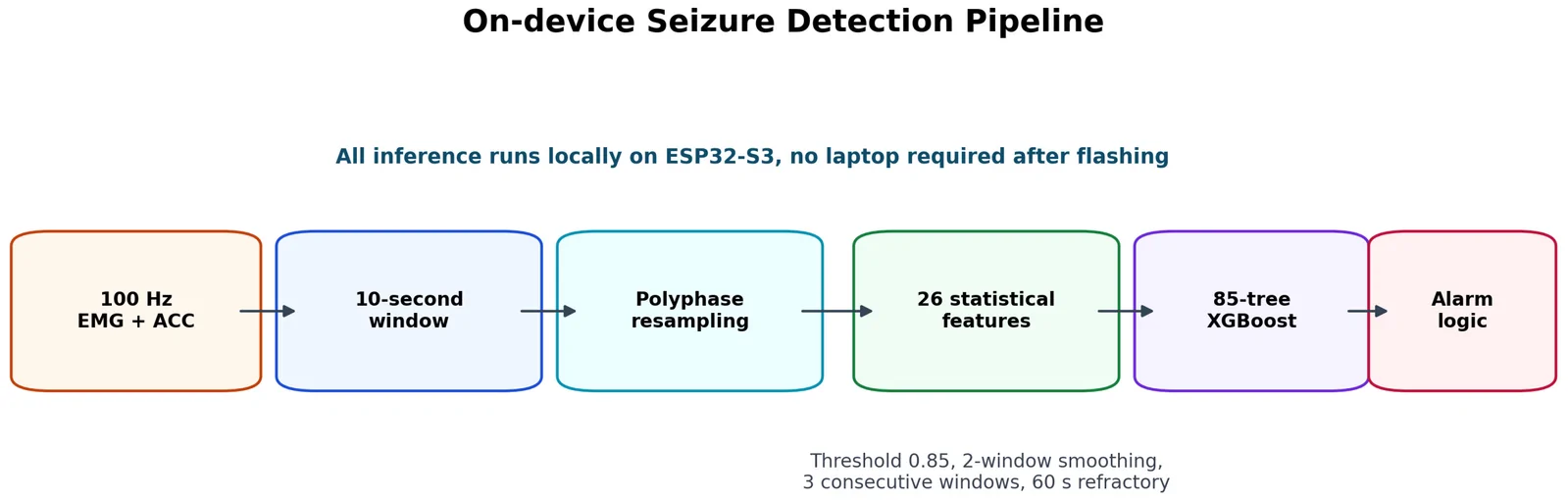
On-device seizure-detection pipeline used by the deployed firmware, from 100 Hz EMG/acceleration acquisition to the 85-tree XGBoost classifier and alarm logic.

The implementation-focused on-device pipeline summarizes the timing of the deployed detector: synchronized 100 Hz EMG/acceleration acquisition, 10 s windows, resampling, 26 statistical features, an 85-tree XGBoost classifier, and temporal alarm logic. All inference is performed locally on the ESP32-S3 after firmware flashing. The complete on-device seizure-detection sequence is shown in Figure 11.

#### 3.6.4. Alarm Decision Parameters

The final decision profile used a seizure-probability threshold of 0.85. Probability estimates were smoothed over two windows, and three consecutive qualifying windows were required before alarm activation. A 60 s refractory interval limited repeated alarms, and local buzzer/vibration output was maintained for 10 s. These settings intentionally trade faster response for resistance to isolated high-probability windows and should be interpreted as deployment settings rather than a clinically optimized operating point. Table 7 summarizes the final alarm-decision parameters implemented in the firmware.

The threshold and temporal parameters are deployment settings selected to suppress isolated high-probability activations and stabilize alarms; they are not presented as a clinically optimized operating point. The available source record does not contain a formal sweep of probability threshold, window length/overlap, smoothing length, or confirmation count. A future ablation should vary these parameters jointly and report sensitivity, false alarms per 24 h, precision/F1, and detection latency for each operating point.

The decision rule can be stated explicitly. Let *p*_k_ be the classifier probability for window *k* and $\bar{p}$_k_ = (*p*_k_ + *p*_k−1_)/2 the two-window smoothed probability. An alarm is issued at the end of window *k* when $\bar{p}$_j_ ≥ 0.85 for *j* = *k* − 2, *k* − 1, and *k*, and no alarm has been issued during the preceding 60 s refractory interval. Because each decision requires a completed 10 s window and three consecutive qualifying windows, the earliest possible confirmed alarm occurs approximately 30 s after the start of the first qualifying window. This structural delay can increase by up to one further window when seizure onset falls part-way through a window or when smoothing averages the first ictal window with a preceding non-ictal window. Therefore, approximately 30–40 s of the reported 61.5 s median latency is attributable to the windowing and confirmation structure itself, which identifies shorter or overlapping windows and fewer confirmation windows as the main levers for reducing latency in future work.

#### 3.6.5. Analytical Ablation of the Temporal Decision Structure

A retraining-based ablation requires the patient-level training data and protocol, which are not available in the present record (Section 3.8). The effect of the temporal decision structure was therefore analyzed analytically. For a window of length *L*, a window step *s*, and a requirement of *C* consecutive qualifying windows, the earliest confirmed alarm occurs *L* + (*C* − 1)*s* after the start of the first qualifying window, and seizure onset falling part-way through a window can add up to one further step *s*. The number of classifier evaluations per hour is 3600/*s*, which scales the inference workload. Table 8 lists these quantities for the deployed setting and for alternative settings. The values are structural bounds derived from the decision rule, not measured detection results. The effect of each setting on sensitivity and false alarms can only be established by re-evaluation on annotated data, and settings with *L* ≠ 10 s would additionally require retraining because the model was trained on 10 s windows.

Relative to the deployed setting, a 50% window overlap with the same three-window confirmation would lower the structural delay from 30 s to 20 s while doubling the inference rate. Reducing confirmation to a single window gives the shortest delay but removes the protection against isolated high-probability windows that the confirmation rule was designed to provide. Modality-level ablation (EMG-only, acceleration-only, EMG + ACC, and EMG + ACC + PPG) cannot be obtained from the exported model, because the 85 trees were trained jointly on all 26 features and removing one modality’s inputs would not yield a valid single-modality classifier. It requires retraining each configuration under the same patient-level protocol and is specified as stage (3) of the future work in Section 4.10.

#### 3.6.6. Computational Load: Analytical Estimate

The per-window workload of the deployed pipeline can be bounded from its structure. Each 10 s decision processes 1000 EMG samples, interpolated to 2560 values, and 1000 three-axis acceleration samples, reduced to a 250-value magnitude series; 2810 values are therefore summarized per window. Eleven of the 13 statistics (all except the median and interquartile range) require only a fixed number of linear passes over the data, whereas the median and quartiles require ordering, which costs O(*N* log *N*) operations by sorting (about 2.9 × 10^4^ comparisons for *N* = 2560) or O(*N*) by selection. Tree inference requires at most 85·*D* node comparisons, where *D* is the maximum tree depth, followed by 85 additions and one exponential; even at a depth of 10 this is fewer than 10^3^ comparisons. The total is therefore of the order of 10^5^ elementary operations per 10 s window. On an ESP32-S3 core clocked at up to 240 MHz [12], this corresponds to an estimated execution time in the low-millisecond range, well under 1% of the 10 s window. Processing is therefore not expected to constrain real-time operation, and the higher inference rates of the overlapping-window settings in Table 8 would remain feasible. Storing the 2810 resampled values as 32-bit floats would require approximately 11 kB of working memory. These figures are analytical estimates based on operation counts, not measurements. Measured feature-extraction and inference times, RAM and flash use, and current consumption have not yet been obtained, and they are required before resource efficiency can be compared quantitatively with the systems in Table 1.

### 3.7. IoT Communication and Complete System Integration

**Figure 12:**
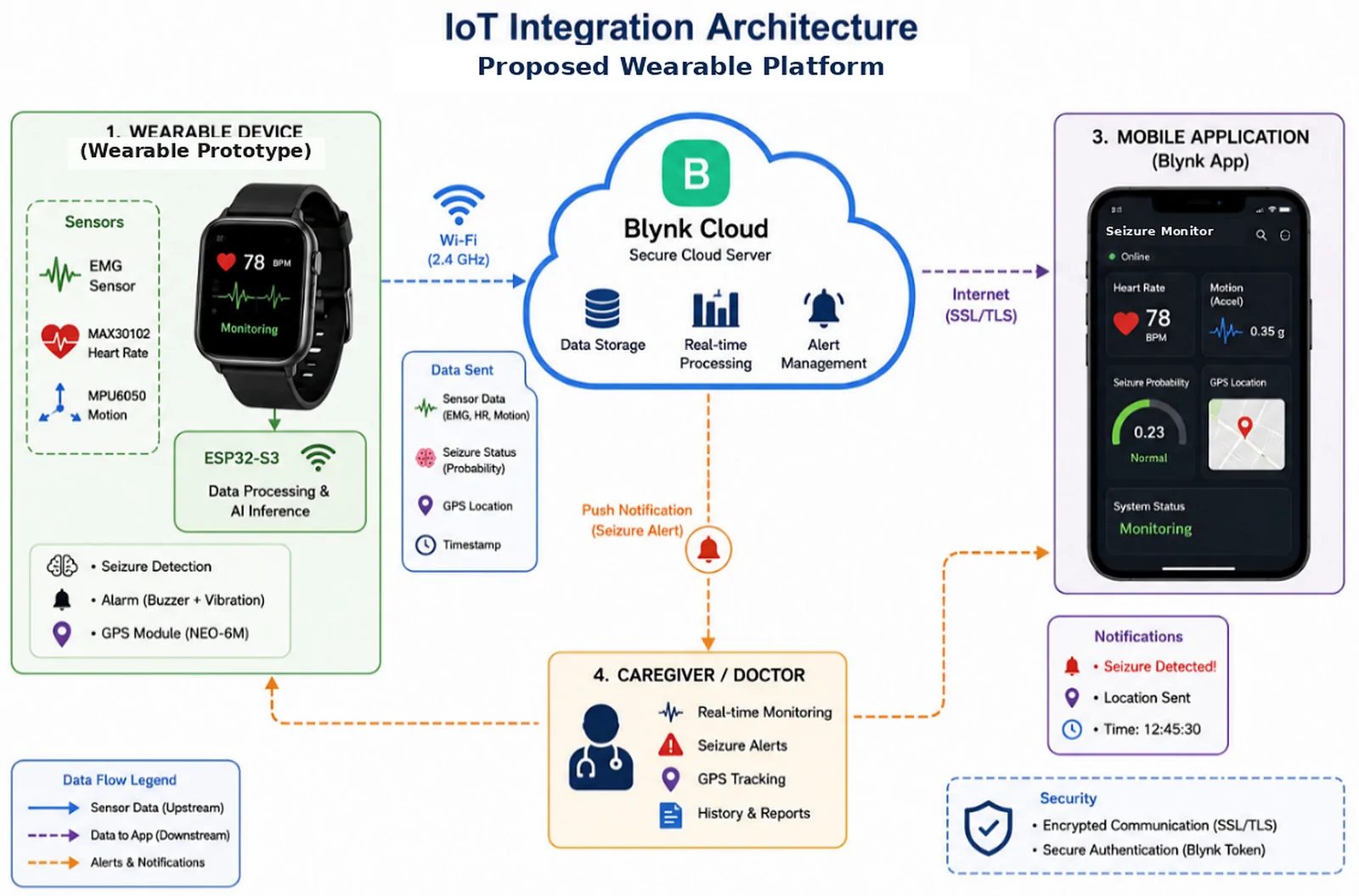
IoT integration architecture linking the wearable device, Blynk Cloud, caregiver/mobile interface, telemetry channels, and alert functions.

The wearable communicates with Blynk Cloud through Wi-Fi [24,25]. Telemetry includes heart rate, raw EMG, motion, AI probability and state, alarm state, GPS/location information, and signal-quality indicators. The mobile interface also supports alarm muting, output testing, and motion-sensor recalibration. Local detection remains independent of cloud connectivity so that loss of the network does not stop embedded inference. Figure 12 illustrates the IoT communication architecture and caregiver-facing data flow.

**Figure 13:**
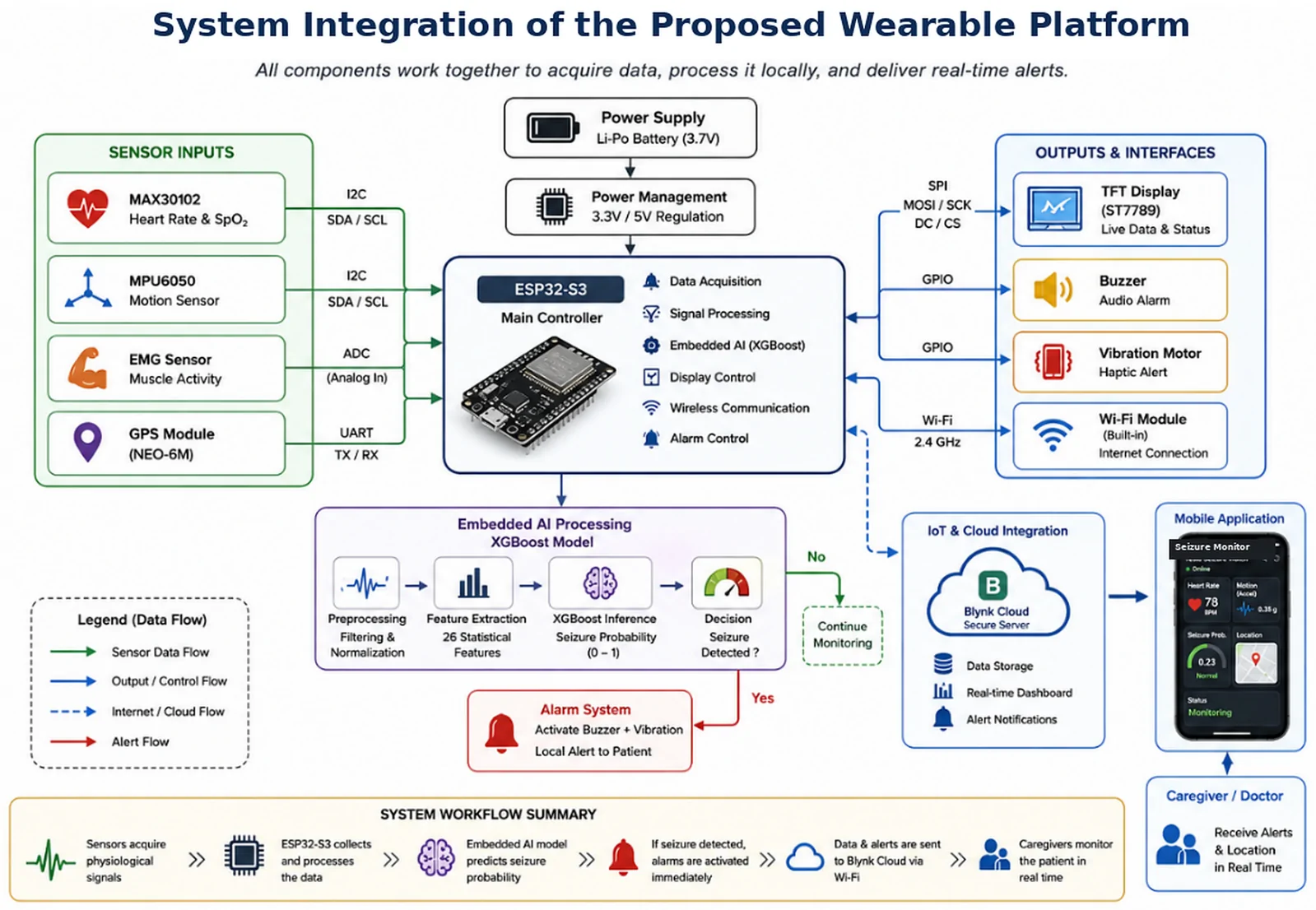
Complete system integration of the proposed wearable platform, showing sensor inputs, ESP32-S3 edge processing, embedded AI, local outputs, IoT communication, and mobile monitoring.

At the complete-system level, all sensing, processing, AI, communication, power, and output subsystems are integrated around the ESP32-S3. The final integration view clarifies the division between input sensors, edge processing, alarm outputs, cloud services, and caregiver-facing interfaces. Figure 13 presents the complete system integration.

### 3.8. Verification and Performance Metrics

Verification was performed at electrical, sensor, module, integration, and user-workflow levels. Electrical checks included power rails, grounding, I^2^C detection, ADC range, and UART wiring. Sensor checks included stationary acceleration, gyroscope bias, PPG response, EMG variation, and GPS data reception. Module-level verification included feature-extraction equivalence, predictor equivalence, and the embedded self-test. Integration testing then evaluated concurrent sampling, interface refresh, Wi-Fi/Blynk communication, alarm outputs, and recovery from unavailable sensors.

For software equivalence, 1000 random 26-feature vectors were evaluated by the embedded predictor and compared with the Python (v 3.10) reference. End-to-end comparisons were also performed on recorded signal windows. Event-level performance was summarized using sensitivity = TP/(TP + FN), precision = TP/(TP + FP), F1-score = 2(precision × sensitivity)/(precision + sensitivity), and false alarms per 24 h = FP/(monitored duration in days). Median detection latency was reported as provided in the available validation record.

Event sensitivity is calculated as Sensitivity = TP/(TP + FN). For the reported record, TP = 12 and FN = 6, giving 12/(12 + 6) = 0.667, reported here as 12/18 (67%) rather than with two-decimal precision. Detection latency is defined operationally as the elapsed time from the reference seizure onset to the first confirmed detector alarm under the implemented temporal decision rule. Because the available validation record supplies the median latency but not the complete event-level onset/alarm timestamp set, the reported 61.5 s median is reproduced descriptively rather than recomputed from individual events.

The temporal decision path uses 10 s non-overlapping windows, two-window probability smoothing, and three consecutive qualifying windows before confirmation. These settings can add algorithmic delay before the first confirmed alarm and are therefore considered when interpreting the 61.5 s median detection latency.

The evaluation duration (2643.69 h), 18 record-labeled FBTCS events, 12 detected events, reported false-alarm rate of 0.31 per 24 h, and median latency of 61.5 s were taken from the available validation record. The present manuscript does not provide a patient-level evaluation split, synchronized video–EEG reference description, or the original event-matching tolerance used to create that record. Therefore, these event-level values are reported descriptively and are not presented as a new prospective clinical validation of the custom wearable hardware.

The available record does not provide the number of unique patients contributing to the reported event-level summary, the full recording count, the patient-level train/validation/test allocation, or the complete non-seizure-period partition. The manuscript therefore reports only the supported totals (2643.69 h, 18 record-labeled FBTCS events, 12 detected events) and does not infer missing cohort structure. Reproduction of the original training stage will require release of the patient-level split, class-balancing procedure, complete XGBoost hyperparameters, random seed, early-stopping rule, and event-matching protocol.

The available documentation also does not state whether all 18 record-labeled target events were independently confirmed as focal-to-bilateral tonic–clonic seizures according to ILAE criteria, whether additional seizure types were present or excluded, or who performed the event labeling/adjudication. Consequently, this revision uses the term “record-labeled FBTCS events” and does not treat the 18 events as independently verified clinical ground truth. A future prospective study must define the seizure taxonomy, inclusion/exclusion rules, expert labeling procedure, clinical reference standard, and event-matching tolerance before analysis.

### 3.9. Reproducibility Package and Public Release

The main project thesis documents a modular software package comprising the main Arduino sketch (currently named Tesla_Watch_AI_V10_PREMIUM_UI.ino), fbtcs_features.cpp/.h for resampling and 26-feature extraction, fbtcs_inference.cpp/.h for tree traversal and logistic probability calculation, fbtcs_model_data.h for the exported tree structure, resample_filters.h for fixed resampling coefficients, and V10_VALIDATION.json for structural/host-side validation. These files, together with the training/feature-extraction scripts and a machine-readable description of the validation record, are required for independent reproduction. Credentials stored in secrets.h must not be released; a credential-free configuration template should be provided instead.

To make the reproducibility status explicit, Table 9 lists each element of the pipeline, where it is documented, and whether it can currently be reproduced from the manuscript. All elements needed to re-implement the deployed inference path (acquisition parameters, resampling, formal feature definitions, the inference equation, and the alarm rule) are now specified in the text. The training stage cannot be reproduced until the items marked as not available are released.

## 4. Results and Discussion

### 4.1. Hardware Integration and Practical Wearability Demonstration

**Figure 14:**
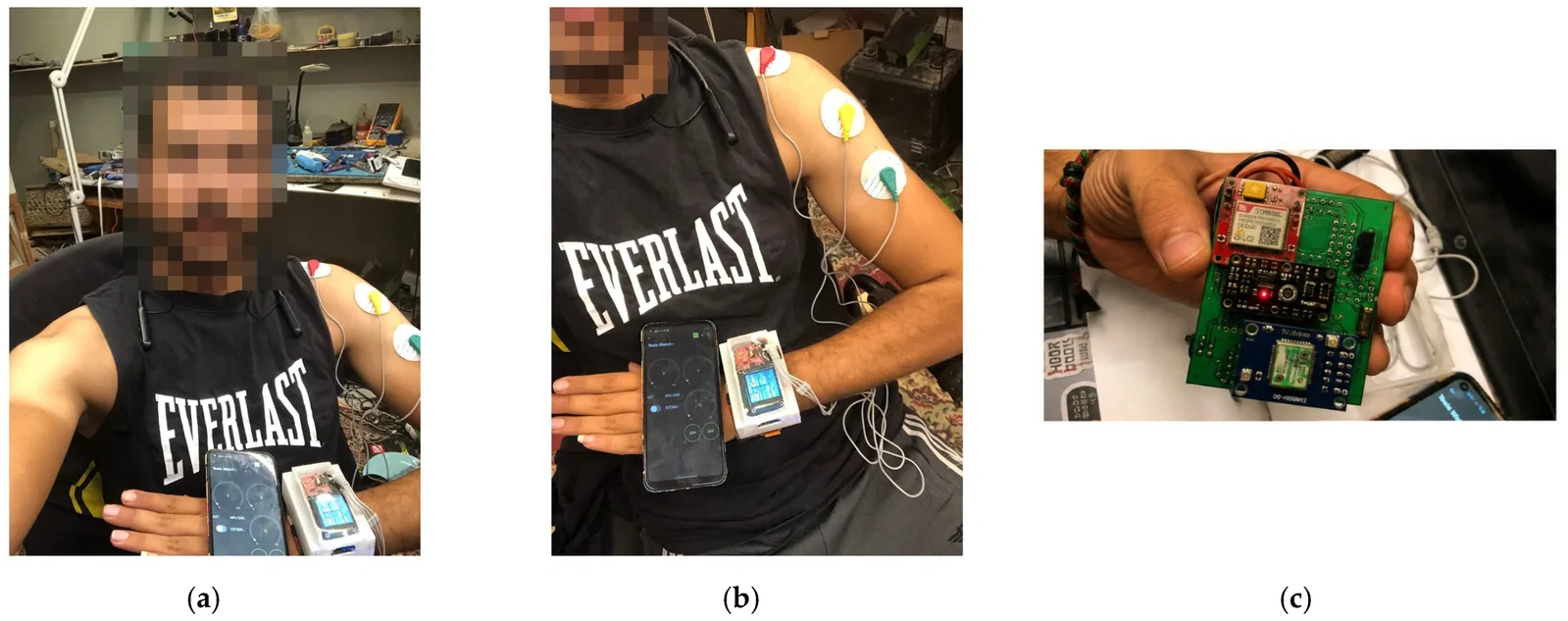
Practical wearability testing of the proposed system: (**a**) full setup with upper-arm electrodes and mobile monitoring; (**b**) close-up of electrode placement, wearable prototype, and mobile interface; (**c**) internal electronic assembly of the wearable prototype. Facial regions in panels (**a**) and (**b**) have been mosaic-processed to protect the identity of the participant.

Functional testing confirmed successful communication among the ESP32-S3, motion sensor, PPG sensor, EMG channel, GPS subsystem, TFT interface, buzzer, vibration motor, and Blynk telemetry. The practical setup demonstrated simultaneous electrode connection, wearable electronics, local display operation, and mobile monitoring. Figure 14 documents the wearability test and the internal electronic assembly.

Panel (c) documents an internal prototype assembly used during hardware development. The final manuscript describes Wi-Fi/Blynk as the primary remote-telemetry path; therefore, components visible in earlier internal assemblies should be interpreted as development-stage hardware documentation rather than evidence that every module remained active in the final firmware revision. The wearability images in Figure 14 are functional device-placement demonstrations and were not used to establish the reported event-level seizure-detection performance.

### 4.2. Embedded Software Equivalence

The embedded feature extractor and predictor reproduced the Python reference with very small numerical error. Across 1000 random feature vectors, the maximum absolute probability difference was approximately 1.19 × 10^−7^. End-to-end validation across recorded windows produced a maximum absolute probability difference of approximately 1.49 × 10^−8^. These results establish numerical software equivalence between the embedded and reference implementations; they do not establish clinical equivalence of the custom sensors.

### 4.3. Event-Level Seizure-Detection Performance

**Figure 15:**
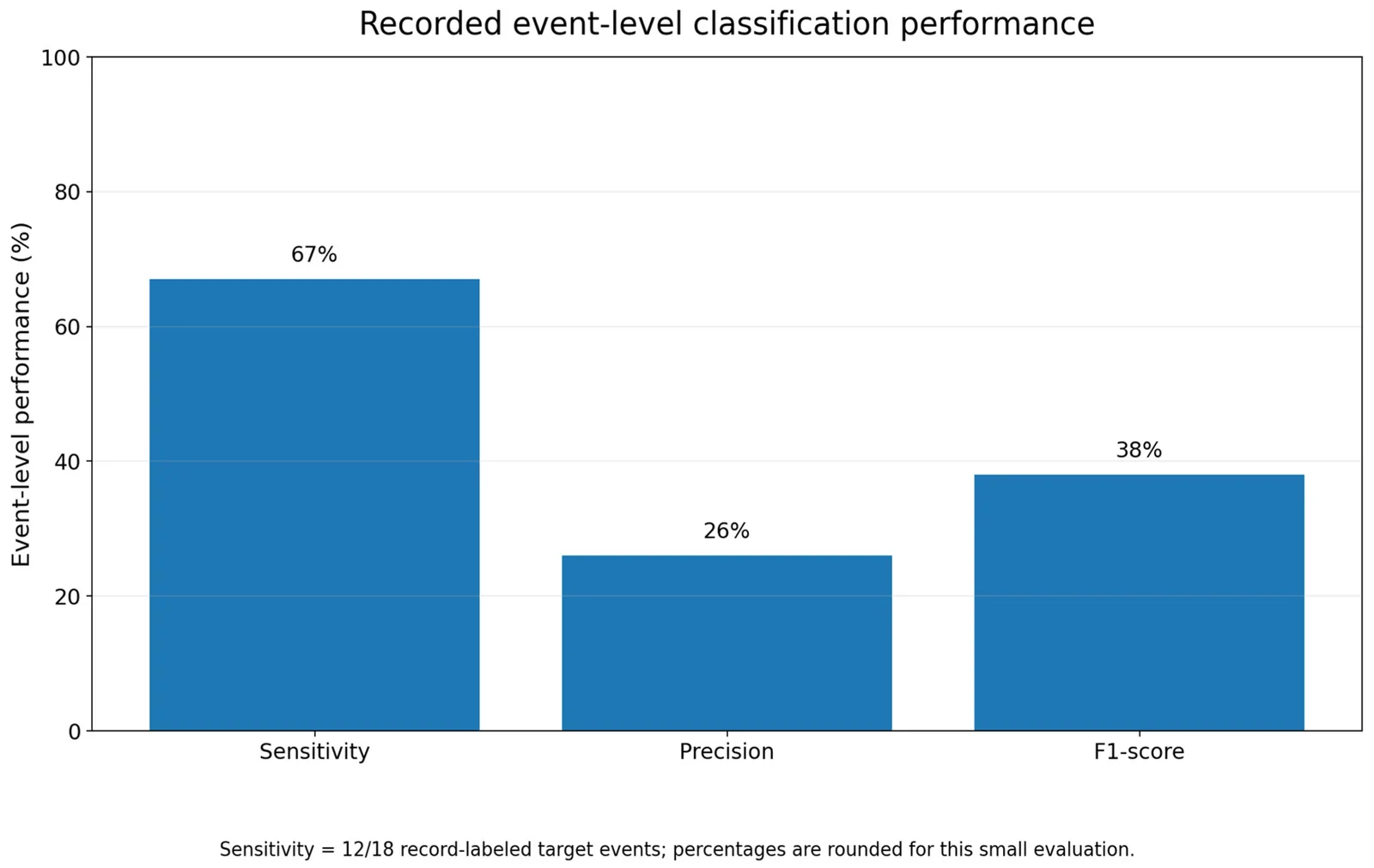
Recorded event-level sensitivity, precision, and F1-score for the proposed detector.

The reported validation record covered 2643.69 h (approximately 110.15 days) and contained 18 record-labeled FBTCS events. Twelve events were detected and six were missed, giving 12/18 (67%) event sensitivity. The Wilson 95% confidence interval is approximately 44–84%, illustrating the substantial uncertainty associated with the small event count. Event precision was approximately 26% and the F1-score approximately 38%. The reported false-alarm rate was 0.31 per 24 h, and median detection latency was 61.5 s. Figure 15 summarizes the principal event-level classification metrics, while Table 10 consolidates the values and identifies whether each quantity was directly reported or derived.

Using the reported false-alarm rate and total duration gives approximately 34 false alarms over the evaluation period. With 12 detected target events, this implies about 46 total alarms, of which approximately 74% were false, or about 2.8 false alarms for every detected seizure alarm. Such a burden can create caregiver alarm fatigue and is incompatible with a claim of present clinical readiness despite the apparently modest normalized rate of 0.31 false alarms per 24 h. The approximately 34 false alarms are a post hoc estimate from rate × duration, not a directly observed count in the available record.

To quantify uncertainty in the remaining metrics, the same methods were applied to the derived counts. Treating the 46 estimated alarms as binomial trials, the Wilson 95% confidence interval for precision (12/46 ≈ 26%) is approximately 16–40%. Treating the ≈34 estimated false alarms over 110.15 days as a Poisson count, the exact 95% confidence interval for the false-alarm rate is approximately 0.21–0.43 per 24 h. Because both intervals rest on the estimated rather than directly observed false-alarm count, they are reported as indicative ranges only.

The 18-event evaluation is too small to support strong clinical-efficacy conclusions or reliable patient-level generalization. It is treated here as pilot evidence that quantifies uncertainty and can help plan the sample size and statistical power of a future prospective, patient-level validation study.

**Figure 16:**
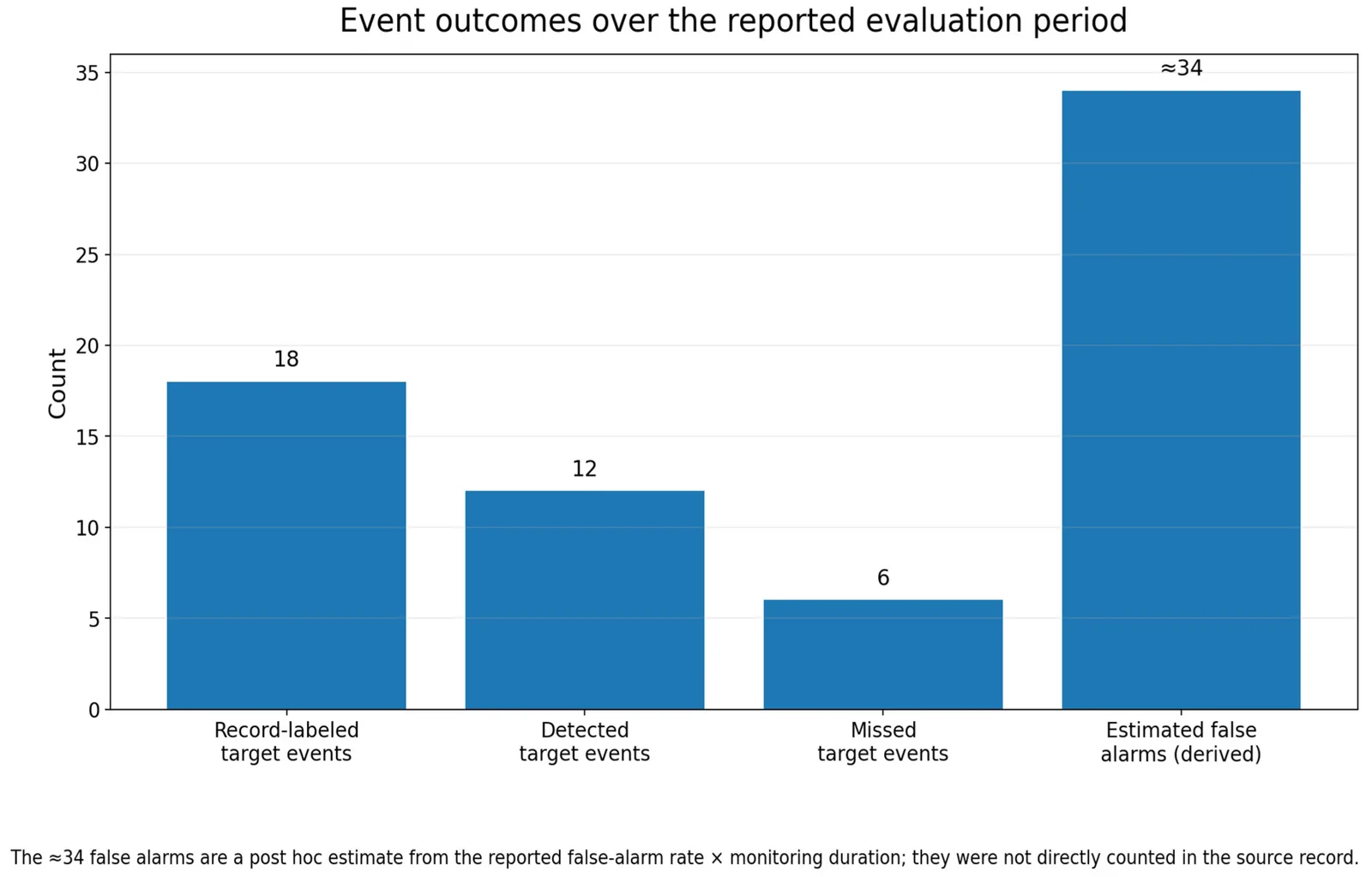
Event outcomes across the reported evaluation. A false alarm is an alarm generated by the system when no actual seizure event occurred. The false-alarm count (≈34) is an estimated count derived from the reported false-alarm rate and total evaluation duration, not a percentage or a directly observed count.

The reported false-alarm rate was 0.31 false alarms per 24 h, where a false alarm is defined as an alarm generated by the system in the absence of an actual seizure event. Figure 16 summarizes the resulting event outcomes: 18 record-labeled target events, 12 detected events, 6 missed events, and the estimated false-alarm count (≈34).

### 4.4. IoT and System-Level Functional Validation

During functional testing, Wi-Fi connection, Blynk connection, sensor-data upload, GPS transmission, local alarm activation, and notification delivery were all reported as successful. The mobile dashboard remained synchronized with the wearable during testing, and remote commands such as alarm muting, alarm testing, and sensor recalibration could be executed without interrupting local seizure inference.

### 4.5. Performance Context Relative to Prior Wearable Tonic–Clonic Seizure Detectors

**Figure 17:**
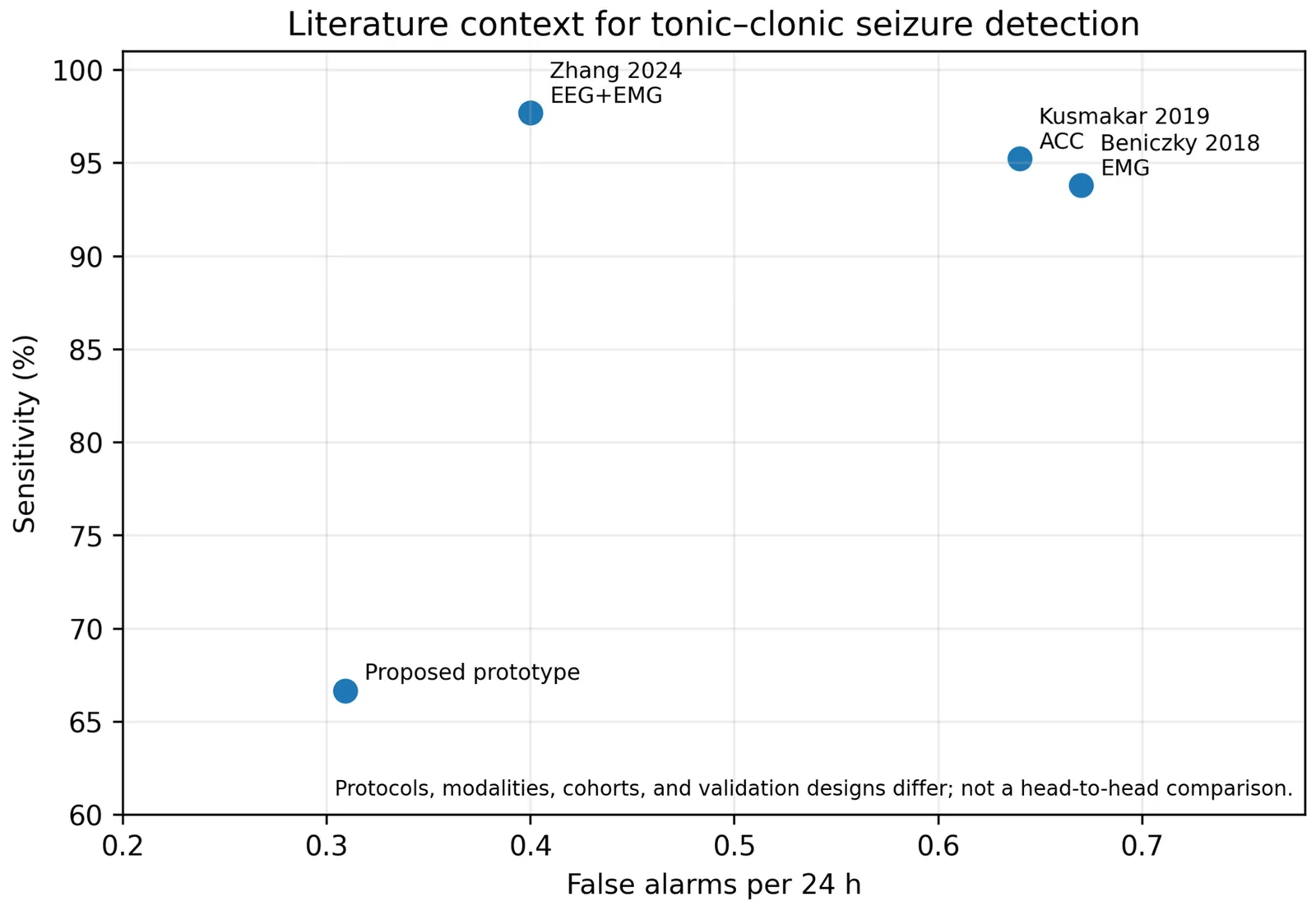
Descriptive literature context for sensitivity and false-alarm burden. Values are not directly comparable because study protocols, populations, modalities, and reference standards differ [5,6,10].

Figure 17 places the proposed system’s reported sensitivity and false-alarm burden beside representative published wearable tonic–clonic/convulsive-seizure detectors reviewed in Section 2. This comparison is descriptive rather than a head-to-head benchmark because sensor modalities, patient populations, event definitions, monitoring environments, and reference standards differ across studies [5,6,7,8,10].

### 4.6. Principal Findings

This study demonstrates the engineering feasibility of running an end-to-end multimodal seizure-detection pipeline on a resource-constrained microcontroller-class wearable platform. The main technical success is not a high clinical classification score; rather, it is the integration of deterministic acquisition, fixed feature generation, a nontrivial boosted-tree model, local decision logic, a responsive interface, and IoT telemetry without requiring a cloud inference service. Numerical agreement with the reference Python model to approximately 10^−7^–10^−8^ confirms that moving the trained classifier onto the ESP32-S3 did not materially alter its probability calculation.

The event-level results reveal a more cautious picture of detection performance. Sensitivity of 12/18 (67%) means that one-third of the record-labeled FBTCS events were missed. The corresponding Wilson 95% confidence interval is broad (approximately 44–84%) because only 18 target events were represented. Precision of approximately 26% means that most alarmed events were not target seizures; the derived alarm composition indicates that about 74% of alarms were false. Both metrics matter: a detector can have a modest daily false-alarm rate while still producing low precision when the target event is rare. The approximately 38% F1-score reflects this joint limitation.

Median detection latency of 61.5 s is also clinically important. The current implementation requires 10 s non-overlapping windows, probability smoothing, and three consecutive qualifying windows, so the temporal logic inherently introduces delay. The reported latency therefore supports the term “detection” rather than “prediction.” No pre-ictal horizon, seizure-prediction characteristic, or negative-latency endpoint was reported. A future prediction claim would require a prospectively defined seizure occurrence period and prediction horizon distinct from event detection.

### 4.7. False-Alarm/Sensitivity Trade-Off

Wearable seizure detectors must balance missed events against nuisance alarms. The ILAE/IFCN guideline emphasizes use of clinically validated devices for tonic–clonic seizure detection and the importance of practical false-alarm burden [3]. The 2022 meta-analysis by Naganur et al. found high average sensitivity across published tonic–clonic detection studies but also substantial between-study heterogeneity and persistent false-alarm concerns [4]. In this study, a false alarm refers to an alarm generated by the detector when no actual seizure event occurred. The proposed detector produced a reported false-alarm rate of 0.31 false alarms per 24 h.

The threshold and confirmation settings provide a plausible explanation for part of this trade-off. A high probability threshold (0.85), two-window smoothing, three-window confirmation, and a 60 s refractory interval favor alarm specificity and stability at the expense of rapid response and sensitivity to short or atypical events. Future optimization should therefore evaluate threshold, window length, overlap, smoothing, and confirmation count jointly rather than tuning model probability alone. Patient-specific calibration may also be important because muscle activation and movement patterns differ across individuals.

The lower sensitivity should not be attributed to thresholding alone. First, the model was developed from SeizeIT2-derived signals whereas the custom device uses a different electrode/sensor chain, creating dataset-to-hardware domain shift. Second, hardware EMG is sampled at 100 Hz and then interpolated to 256 Hz; interpolation does not restore information above the 50 Hz Nyquist limit and therefore cannot make the signal spectrally equivalent to a native higher-rate recording. Third, upper-arm electrode placement, analog-front-end behavior, contact quality, and patient-specific muscle recruitment can shift the feature distribution relative to the reference data. Fourth, conservative smoothing/confirmation logic can miss short or borderline events. Finally, only 18 record-labeled FBTCS events are represented, so 12 detections yield a wide Wilson 95% confidence interval (approximately 44–84%). These factors together provide a more objective explanation for the reported 67% sensitivity and motivate hardware-rate-matched retraining and larger prospective validation.

### 4.8. Multimodality and Edge Deployment

Multimodal sensing is a strength of the hardware platform, but not all available channels currently contribute to the classifier. The decision path uses EMG and acceleration; PPG, gyroscope, and GPS are contextual. Prior work suggests that fusing complementary channels can reduce false positives because the modalities respond differently to movement, autonomic change, and cerebral activity [7,8,9,10]. Zhang et al., for example, reported a substantial reduction in false-positive rate when wearable EEG was fused with EMG and/or accelerometry [10]. The present architecture can therefore be viewed as a modular platform for future sensor fusion rather than a final multimodal decision model.

The absence of EEG is a deliberate engineering simplification but also a detection limitation. Wearable EEG provides information closer to the cerebral seizure source, whereas EMG and acceleration primarily reflect peripheral motor manifestations. Published multimodal work shows that adding wearable EEG to EMG/accelerometry can improve false-positive discrimination [10]. A non-EEG design can reduce electrode count, hardware burden, and signal-processing complexity, but it may be less informative for seizures with weak or atypical peripheral motor expression. The present results therefore should not be interpreted as equivalent to EEG-supported classifiers.

Local XGBoost inference has several practical advantages. Tree traversal is deterministic and computationally light for a 26-element feature vector; the model remains operational without internet access; and only compact telemetry needs to be transmitted. This is consistent with the broader rationale for edge health monitoring, where continuous cloud streaming of raw biosignals may be undesirable because of bandwidth, power, privacy, and connectivity constraints. The ESP32-S3’s integrated wireless and peripheral capabilities make it a reasonable prototyping platform for such edge applications [12].

No complete ablation study is available in the supplied validation record for window parameters, probability threshold, confirmation count, or modality contribution. The current classifier uses EMG + acceleration only; PPG and gyroscope are contextual channels. A reproducible modality ablation should compare EMG-only, acceleration-only, EMG + acceleration, and then EMG + acceleration augmented by PPG and gyroscope using identical patient-level splits and event-matching rules. Likewise, the present embedded-versus-Python verification establishes numerical probability equivalence, but it does not establish seizure-event-level equivalence on clinically labeled data acquired directly from the target hardware. Both experiments are required before stronger claims about multimodal benefit or hardware equivalence are made.

For sensor-level validation, future testing should acquire synchronized target-hardware signals with a clinical reference, quantify feature-distribution differences before and after preprocessing, and evaluate hardware-rate-matched retraining. This would directly test the effect of sensor front end, placement, sampling rate, and preprocessing on event-level outcomes.

### 4.9. Limitations

- The reported event-level performance was taken from the available validation record and should not be interpreted as clinical certification or prospective clinical validation of the custom wearable.
- The manuscript does not report a new prospective patient trial using the custom sensor hardware with synchronized video–EEG as a clinical reference standard. Generalization from SeizeIT2-derived model development to custom sensors therefore remains unproven.
- The deployed hardware acquires EMG at 100 Hz and interpolates it to 256 Hz for reference-pipeline compatibility. This interpolation does not restore higher-frequency signal content and may introduce a sampling-domain mismatch relative to native higher-rate training data.
- The complete original model-training configuration—including the patient-level split, class-balancing strategy, full XGBoost hyperparameters, random seed, and early-stopping criterion—is not reported in this manuscript, limiting independent reproduction of the training stage even though embedded inference equivalence is documented.
- Only 18 record-labeled FBTCS events were represented in the reported evaluation summary; the resulting 12/18 (67%) sensitivity estimate has a wide Wilson 95% confidence interval (approximately 44–84%) and cannot characterize patient-to-patient variability. The event-label provenance and clinical adjudication procedure are also incompletely documented.
- The 61.5 s median detection latency is too long for many rapid-response applications and is partly driven by 10 s windows and multi-window confirmation.
- The derived alarm composition indicates that approximately 74% of generated alarms were false (about 2.8 false alarms per detected target event). This is a substantial practical limitation for caregiver use and reinforces that the current detector is an engineering proof-of-concept rather than a clinically acceptable alarm system.
- The hardware architecture was not selected through a formal comparative optimization study. In particular, the mature MPU6050 and the AD8232-based analog front end were implementation choices; modern alternatives were not benchmarked in the available project record.
- PPG, gyroscope, and GPS are presently contextual rather than classifier inputs, so the term multimodal refers to the platform architecture while the trained seizure decision itself is bimodal (EMG + acceleration).
- Embedded inference time, processor utilization, RAM/flash usage, power consumption, battery runtime, bill-of-materials cost, enclosure ergonomics, wireless cybersecurity, long-term electrode wearability, and sensor-to-reference calibration were not quantitatively characterized. The present engineering claim is therefore limited to functional edge feasibility rather than demonstrated cost, energy, or resource superiority.
- Accordingly, terms such as “AI-efficient” or “low-power” are not used as quantified performance claims for the present prototype. The demonstrated result is that the complete feature-extraction/XGBoost/alarm pipeline runs locally on the ESP32-S3. Quantitative efficiency claims will require measured inference time, processor utilization, model/RAM/flash footprint, average and peak current, energy per decision, and battery runtime under a defined workload.
- The current work reports event detection; it does not validate pre-ictal seizure prediction or forecasting.

### 4.10. Priorities for Clinical Translation

A stronger next-stage study should use prospective, patient-level validation with synchronized video–EEG as the reference, a pre-registered matching rule for event detection, leave-one-patient-out or fully held-out subject evaluation, and confidence intervals for sensitivity and false-alarm rate. Training and evaluation should also reproduce the deployed sensor bandwidth and sampling chain, or retrain the model using hardware-rate-matched preprocessing, to reduce dataset-to-device domain shift. Overlapping windows or a shorter first-stage detector could reduce latency, while a second-stage confirmation model could preserve false-alarm control. Sensor fusion with PPG/heart-rate dynamics, gyroscope features, and potentially electrodermal activity or wearable EEG should be evaluated using ablation studies. Finally, the device should be characterized for inference latency, memory use, power and battery life, thermal behavior, electrode comfort, wireless failure modes, encrypted telemetry, and safe alert escalation before any unsupervised use.

Before enrollment, the prospective protocol should predefine clinically justified target ranges for event sensitivity, precision, false alarms per 24 h, and detection latency, together with stopping/acceptance criteria. This prevents post hoc interpretation of whichever metrics happen to be favorable and provides a clear benchmark for clinical utility.

Future work will therefore proceed in four linked stages: (1) release/reconstruct a fully reproducible patient-level training and evaluation protocol; (2) collect prospective target-hardware data synchronized with video–EEG and quantify sensor/preprocessing domain shift; (3) perform threshold, temporal-decision, sampling-rate, and modality ablations; and (4) benchmark inference time, memory use, power/energy, battery life, cost, usability, and alert reliability. These steps are necessary before the platform can be assessed as a clinically deployable wearable rather than an engineering prototype.

## 5. Conclusions

A complete multimodal wearable seizure-monitoring prototype was implemented around the ESP32-S3 with synchronized EMG and inertial acquisition, embedded 26-feature extraction, an 85-tree XGBoost classifier, local TFT/buzzer/vibration outputs, GPS context, and Blynk IoT connectivity. Embedded inference reproduced the Python reference to within approximately 10^−7^–10^−8^ probability error. In the reported validation record covering 2643.69 h, 12 of 18 record-labeled FBTCS events were detected (67% sensitivity; Wilson 95% CI approximately 44–84%), with approximately 26% precision, approximately 38% F1-score, a reported 0.31 false alarms per 24 h, and a median detection latency of 61.5 s. The estimated alarm composition implies that roughly 74% of alarms were false. Together with the small event count and incomplete clinical-label provenance, these results support engineering feasibility only; they do not constitute prospective clinical validation and do not support clinical or unsupervised emergency deployment. The most important next steps are complete public reproducibility, patient-level clinical validation with a defined reference standard, improved sensitivity/precision/latency, hardware-rate-matched model development, sensor-fusion ablation, and quantitative evaluation of embedded resource use, battery performance, usability, and safety.

## Figures and Tables

**Table 1:** Engineering-oriented comparison with recent edge/MCU seizure-detection implementations. NR = not reported in the present manuscript/source record.

Study	Platform	Model/Sensing	Model/Memory	Inference/Latency	Power/Energy	Engineering Relevance
Qian et al. [14]	MSP432 MCU	Compressed learning + 1-D CNN; EEG	50.13 kB memory	21.07 ms/classification	13.4727 mW; 269.4 μJ/classification	Direct MCU seizure-detection benchmark
Ingolfsson et al. [15]	GAP9 MCU	BrainFuseNet; EEG + PPG + ACC	NR here	Embedded edge deployment	0.11 mJ/inference (high-performance point)	Ultra-low-power multimodal edge deployment
Hizem et al. [16]	Microcontroller-class TinyML	ExtraTrees/TinyML; EEG	256 kB optimized model	Reduced after conversion; exact device benchmark not used here	Energy-efficient TinyML focus	Recent compact-model reference
Busia et al. [17]	Apollo4, GAP8, GAP9 MCUs	EEGformer compact transformer; temporal-channel EEG	NR here	13.7 ms/inference (GAP9)	0.31 mJ/inference (GAP9)	MCU deployment with event-level reporting (0.15 FP/h on CHB-MIT)
Li et al. [18]	Xylo SNN neuromorphic processor	Spiking neural network; EEG	NR here	Real-time, low-latency (reported)	87.4 μW I/O + 287.9 μW compute	Sub-milliwatt always-on detection
Zanghieri et al. [19]	Low-power parallel MCU	Temporal convolutional network; iEEG	NR here	5.68 ms (1 core); 1.46 ms (8 cores)	124.5 μJ (1 core); 51.2 μJ (8 cores)	Per-inference latency/energy benchmark
Popa et al. [20]	ESP32 (520 KiB RAM, 240 MHz)	Edge Impulse model, int8; EEG	NR here	15 ms/inference (int8)	NR	Same MCU family as the proposed platform
Proposed system	ESP32-S3	85-tree XGBoost; EMG + ACC decision path	Not quantitatively characterized	Event-level median detection latency 61.5 s; pure inference time NR	Not quantitatively characterized	Functional embedded sensing-to-alert feasibility; resource benchmarking remains future work

**Table 2:** Comparative summary of the reviewed wearable seizure-detection literature and the proposed system.

Study/System	Sensors and Algorithm	Validation and Reported Performance	Main Contribution, Limitation, and Relation to Proposed System
Beniczky et al. (2018) [5]	Surface EMG; real-time wearable algorithm	Prospective GTCS evaluation: 93.8% sensitivity; 0.67 false alarms/24 h; detection within 30 s.	Strong single-modality performance; electrode placement/contact and muscle-specific artifacts remain limitations. The proposed system retains EMG but adds acceleration and edge integration.
Kusmakar et al. (2019) [6]	Wrist accelerometer; time-domain/Poincaré features + SVDD	5576 h, 79 patients. GTCS subgroup: 95.23% sensitivity; 0.64 false alarms/24 h.	Simple and unobtrusive, but vigorous daily activity can mimic convulsive motion. This motivates the proposed EMG + ACC fusion.
Onorati et al. (2017) [7]	Wrist ACC + EDA; multimodal machine learning	Multicenter evaluation: 52/55 convulsive seizures detected (~94.5%); ~0.2 false alarms/24 h.	Demonstrated benefit of combining motor and autonomic information; greater sensor complexity than unimodal approaches.
Onorati et al. (2021) [8]	Wrist ACC + EDA; fixed-and-frozen algorithm	Prospective multicenter video–EEG: 152 patients, 429 days, 66 seizures. Sensitivity 0.92 pediatric/0.94 adult; FAR 1.26/0.57 per 24 h.	Strong phase-III-style validation on independent patients; performance varied with activity and age. The proposed system has not yet reached this validation level.
Zhang et al. (2024) [10]	Behind-ear EEG + EMG + ACC; multimodal fusion	27 participants, 44 TCS. EEG + EMG + ACC: 90.9% sensitivity; 0.1 FP/24 h; 75.5% precision; 82.5% F1.	Excellent multimodal discrimination; wearable EEG adds hardware and signal-processing complexity relative to the microcontroller-based proposed system.
Bhagubai et al. (2025), SeizeIT2 [11]	Open wearable EEG, ECG, EMG, ACC and gyroscope dataset	~11,640 h of multimodal recordings; dataset resource rather than a standalone detector-performance study.	Enables reproducible algorithm development, but does not validate a custom wearable device by itself.
Proposed system	EMG + ACC decision path; ESP32-S3 embedded XGBoost; PPG/GPS context; IoT	Reported validation record: 2643.69 h; 18 record-labeled FBTCS events. 12/18 detected (67% sensitivity); 0.31 false alarms/24 h; ≈26% precision; ≈38% F1; 61.5 s median latency.	Fully embedded microcontroller-class sensing-to-alert architecture; sensitivity and latency require substantial improvement and prospective clinical validation.

ACC, accelerometry; EDA, electrodermal activity; FAR, false-alarm rate; FP, false positive; GTCS, generalized tonic–clonic seizure; SVDD, support vector data description; TCS, tonic–clonic seizure. Reported values are descriptive and are not directly comparable across studies because cohorts, sensor placement, seizure definitions, reference standards, and evaluation protocols differ.

**Table 3:** Main proposed-system hardware and assigned functions.

Component	Interface/Signal	Function in Prototype
ESP32-S3	ADC, I^2^C, UART, SPI, Wi-Fi	Central acquisition, processing, embedded inference, UI, and IoT communication
Analog EMG front end (AD8232-based [26])	Analog → GPIO4 ADC	Muscle biopotential acquisition used by classifier
MPU6050	I^2^C	3-axis acceleration and gyroscope; acceleration used by classifier
MAX30102	I^2^C/FIFO	PPG and heart-rate context
NEO-6M GPS	UART	Location context for remote alerting
ST7789 TFT	SPI	Local physiological/system-status interface
Buzzer + vibration motor	Digital outputs via driver stages	Local audible and haptic alerts
Wi-Fi + Blynk Cloud	2.4 GHz Wi-Fi	Remote telemetry, notifications, and control

**Table 4:** Effective sensor modalities and their role in seizure decision.

Sensor/Channel	Acquisition	Classifier Input	Effect on Alarm	Function
AD8232 EMG	100 Hz; interpolated to 256 Hz	Yes (13 features)	Determines alarm via classifier	Primary decision modality
MPU6050 accelerometer	100 Hz, 3 axes → magnitude; resampled to 25 Hz	Yes (13 features)	Determines alarm via classifier	Primary decision modality
MPU6050 gyroscope	100 Hz	No	None	Motion context, display, recalibration
MAX30102 PPG	≈25 effective records/s	No	None	Heart rate, signal-quality state, telemetry
NEO-6M GPS	UART, continuous	No	None	Location context for remote alerts

**Table 5:** Principal firmware data paths and execution rates.

Data Path	Rate/Trigger	Purpose
EMG ADC → sensor task	100 Hz	Seizure features and live waveform
MPU6050 → sensor task	100 Hz	Acceleration window; gyroscope context
MAX30102 FIFO → heart processor	≈25 effective records/s	Heart-rate estimate and signal-quality state
GPS UART → main loop	Continuous	Location, fix state, satellite/time context
AI task → controller	Every 10 s window	Probability, temporal decision logic, alarm trigger
UI timer → ST7789	200 ms	Responsive local display
Cloud timer → Blynk	1 s	Telemetry and remote controls

**Table 6:** Statistical feature set applied to both EMG and acceleration magnitude.

No.	Statistic	Interpretation
1	Mean	Average signal level after preprocessing
2	Standard deviation	Dispersion around the mean
3	Centered RMS	Root-mean-square of the centered signal
4	Median	Robust central value
5	Interquartile range	75th minus 25th percentile
6	Minimum	Minimum sample value
7	Maximum	Maximum sample value
8	Range	Maximum minus minimum
9	Mean absolute deviation	Mean absolute centered amplitude
10	Maximum absolute deviation	Largest centered amplitude
11	Difference RMS	RMS of consecutive-sample differences
12	Line length	Mean absolute consecutive-sample difference
13	Zero-crossing rate	Fraction of sign changes around the mean

**Table 7:** Embedded alarm-decision parameters.

Parameter	Value
Analysis window	10 s
Window step	10 s (non-overlapping)
Probability threshold	0.85
Smoothing	2 windows
Consecutive confirmation	3 windows
Refractory interval	60 s
Local output duration	10 s

**Table 8:** Analytical ablation of window step and confirmation count (*L* = 10 s; *C* = consecutive qualifying windows required). Delays exclude the additional step that two-window smoothing can introduce.

Configuration	Step *s*	*C*	Min. Delay	With Onset Offset	Inferences/h	Workload
Deployed (non-overlapping)	10 s	3	30 s	30–40 s	360	1×
Single-window decision	10 s	1	10 s	10–20 s	360	1×
Two-window confirmation	10 s	2	20 s	20–30 s	360	1×
50% overlap	5 s	3	20 s	20–25 s	720	2×
80% overlap	2 s	3	14 s	14–16 s	1800	5×

**Table 9:** Reproducibility status of the pipeline elements.

Pipeline Element	Where Documented	Status
Hardware interconnection and sensor interfaces	Table 3, Figure 4, Section 3.4.2	Reported
Sampling, buffering, and task rates	Section 3.5.2, Table 5	Reported
Resampling (EMG 100 → 256 Hz; ACC 100 → 25 Hz)	Section 3.6.1	Reported; coefficients in repository
Definitions of the 26 features	Section 3.6.1, Table 6	Reported (formal definitions)
Model structure (trees 0–84 of 165)	Section 3.6.2	Reported
Inference equation	Section 3.6.2	Reported
Alarm decision rule and parameters	Section 3.6.4, Table 7	Reported
Embedded–Python equivalence test	Section 3.8 and Section 4.2	Reported
Firmware, feature code, exported model	Repository (Section 3.9)	To be released with DOI
Training hyperparameters, seed, class balancing, early stopping	—	Not available in source record
Patient-level split and event-matching tolerance	—	Not available in source record
Measured inference time, memory, and power	—	Not yet measured

**Table 10:** Event-level evaluation summary with provenance of reported and derived values.

Metric	Value	Provenance
Record-labeled FBTCS events	18	Source validation record
Detected target events	12	Source validation record
Missed target events	6	Derived: 18 − 12
Event sensitivity	12/18 = 67%	Derived from event counts
Event precision	≈26%	Reported; arithmetic cross-check with estimated FP
Event F1-score	≈38%	Reported/rounded
False-alarm rate	0.31 per 24 h	Source validation record; rounded
Median detection latency	61.5 s	Source validation record
Evaluated duration	2643.69 h (≈110.15 days)	Source validation record
Estimated false-alarm count (not percentage)	≈34	Derived: FAR × duration
Sensitivity 95% CI (Wilson)	≈44–84%	Derived from 12/18

Derived values shown in the figures and table are arithmetic or statistical consequences of the reported event counts, false-alarm rate, and evaluation duration. The sensitivity confidence interval uses the Wilson binomial method. No additional clinical events were inferred or added.

## Data Availability

SeizeIT2 is described by Bhagubai et al. [11], which provides the authoritative dataset-access information. The system-specific firmware, feature-extraction/resampling implementation, embedded XGBoost representation, and validation artifacts are required to be deposited in a public persistent repository before resubmission.

## Abbreviations

The following table lists the abbreviations and technical terms used throughout the manuscript.

Abbreviation	Definition
ACC	Accelerometry/acceleration magnitude
ADC	Analog-to-digital converter
AI	Artificial intelligence
EEG	Electroencephalography
EMG	Electromyography
FBTCS	Focal-to-bilateral tonic–clonic seizure
FAR	False-alarm rate
FN	False negative
FP	False positive
GPS	Global positioning system
IoT	Internet of Things
PPG	Photoplethysmography
TCS	Tonic–clonic seizure
TP	True positive
UART	Universal asynchronous receiver–transmitter
Wi-Fi	Wireless local-area networking
XGBoost	Extreme Gradient Boosting
